# The sRNA LhrC5 and Hfq post-transcriptionally regulate ferritin expression in *Listeria monocytogenes*

**DOI:** 10.3389/fmicb.2026.1771046

**Published:** 2026-06-26

**Authors:** Patrycja Gomza, Katarzyna Ścibek, Magdalena Ładziak, Michał Burmistrz, Eva M. S. Lillebaek, Birgitte H. Kallipolitis, Agata Krawczyk-Balska

**Affiliations:** 1 Department of Molecular Microbiology, Biological and Chemical Research Centre, Faculty of Biology, University of Warsaw, Warsaw, Poland; 2 Department of Biochemistry and Molecular Biology, Faculty of Natural Sciences, University of Southern Denmark, Odense, Denmark

**Keywords:** ferritin, Hfq protein, *Listeria monocytogenes*, post-transcriptional regulation, sRNAs

## Abstract

The RNA chaperone Hfq facilitates base-pairing interactions between small regulatory RNAs (sRNAs) and their target mRNAs in Gram-negative bacteria. In Gram-positive species, however, its analogous matchmaker function remains poorly defined, with the notable exception of the Hfq-dependent sRNA LhrA in the pathogen *Listeria monocytogenes*. This bacterium also encodes the LhrC family of homologous sRNAs, which typically repress gene expression by base-pairing near ribosome-binding sites of target mRNAs. Although LhrC1–5 bind Hfq, their previously characterized regulatory functions are Hfq-independent. Here, we focus on LhrC5, which is uniquely encoded within the operon of ferritin (Fri), an iron-storage protein critical for virulence and stress adaptation. We used a combination of *in vitro* and *in vivo* methods including co-immunoprecipitation, northern blot, EMSA, RNA structure probing, *in vitro* translation, Hfq overexpression, and RNA stability analysis. The *fri* gene is transcribed from one σB-dependent and two σA-dependent promoters. We demonstrate that Hfq specifically promotes the binding of LhrC5—but not the homologous LhrC4—to the ribosome-binding site of the σA1-derived *fri* mRNA, resulting in reduced translation *in vitro*. No interaction between LhrC5 and transcripts originating from the σA2 or σB promoters was detected. Consistent with this specificity, cellular analyses show that LhrC5, but not LhrC1–4, selectively destabilizes the σA1-derived *fri* mRNA under osmotic stress conditions, thereby fine-tuning its expression. However, Hfq was not required for this LhrC5-dependent regulation of *fri* mRNA stability under physiological conditions. Notably, Hfq overexpression during σA1-to-σB switching of the *fri* promoter leads to accelerated decay of both σA1- and σB-derived *fri* mRNAs, consistent with the involvement of Hfq in post-transcriptional downregulation of *fri* expression. Collectively, this study identifies *fri* as a new regulatory target of the LhrC sRNA family member LhrC5 and reveals a previously unrecognized role of Hfq in LhrC5-dependent control. These findings uncover additional complexity in riboregulation in this pathogen, with potential implications for iron homeostasis.

## Introduction

Small regulatory RNAs (sRNAs) are key post-transcriptional regulators of gene expression in bacteria. They play crucial roles in controlling essential biological processes, including stress adaptation, starvation, virulence, and biofilm formation (Waters and Storz, 2009). The most prominent group, trans-encoded sRNAs, regulate gene expression by imperfect base pairing with target mRNAs encoded elsewhere in the genome (Waters and Storz, 2009; Lalaouna et al., 2013). Because of their imperfect complementarity, trans-encoded sRNAs often depend on RNA chaperones, such as Hfq, to facilitate sRNA-mRNA interactions (Kavita et al., 2018). The role of Hfq in post-transcriptional gene regulation is well established in Gram-negative bacteria, where it facilitates sRNA–mRNA base pairing and modulates transcript stability and translation (Kavita et al., 2018). As a homohexameric protein, Hfq adopts a ring-like structure with three distinct RNA-binding surfaces that mediate its matchmaker function (Schu et al., 2015; Santiago-Frangos and Woodson, 2018). In *Escherichia coli* Hfq, three distinct RNA-binding surfaces mediate interactions with different RNA motifs. The proximal face specifically recognizes single-stranded 3′ poly-U tails of Rho-independent terminators in sRNAs (Schumacher et al., 2002), while the distal face preferentially binds AAN motifs within single-stranded RNA regions (Mikulecky et al., 2004; Link et al., 2009; Robinson et al., 2014). The rim surface, which features three critical arginine residues, interacts with A/U-rich single-stranded RNAs (Sauer et al., 2012; Santiago-Frangos and Woodson, 2018). Importantly, the number of arginine residues on the rim surface directly modulates Hfq’s RNA annealing activity in *E. coli* (Panja et al., 2013). While Hfq’s function is well-characterized in Gram-negative bacteria, its role in Gram-positive species remains ambiguous and controversial (Jørgensen et al., 2020; Christopoulou and Granneman, 2022). This is thought to be due to the substantial differences in sequence and properties between Hfq proteins from Gram-positive and Gram-negative species. For instance, the basic rim patch of Hfq in Gram-positive bacteria contains fewer arginines compared to their Gram-negative counterparts. This is clearly exemplified by the presence of only a single arginine in *Listeria monocytogenes* Hfq and the complete absence of rim patch arginines in *Staphylococcus aureus* Hfq (Zheng et al., 2016). As a result, these Hfq variants exhibit markedly reduced RNA annealing activity *in vitro*. Another reason for the elusive role of Hfq in Gram-positive bacteria is its frequently low expression level (Christopoulou and Granneman, 2022). Among Gram-positive species studied to date, *Clostridioides difficile* Hfq is uniquely known to be highly expressed under various conditions, correlating with its well-documented global regulatory function (Christopoulou and Granneman, 2022; Fuchs et al., 2023). Nonetheless, although Hfq-dependent regulation is rarely reported in Gram-positive bacteria, a few examples have been documented. These include the sRNA SpoY, which regulates Spo0A—the master regulator of sporulation—in *C. difficile* (Fuchs et al., 2023), and the sRNA LhrA, which requires Hfq to bind its mRNA targets in *L. monocytogenes* (Nielsen et al., 2010, 2011). Regarding LhrA, its regulatory mechanism resembles that of canonical Hfq-dependent sRNAs in Gram-negative bacteria. Specifically, LhrA represses target mRNA translation by base-pairing with sequences near the ribosome-binding site (RBS), thereby inhibiting translation and promoting subsequent mRNA degradation. Notably, both the base-pairing activity and stability of LhrA are strictly dependent on Hfq (Christiansen et al., 2006; Nielsen et al., 2010, 2011).

LhrA was initially identified alongside LhrB and LhrC1-5 as sRNAs that interact with *L. monocytogenes* Hfq (Christiansen et al., 2006). Like LhrA, LhrC1-5 downregulate specific mRNA targets through base pairing. However, to date, studies have shown that unlike LhrA, LhrC1-5’s regulatory functions are Hfq-independent (Sievers et al., 2014, 2015). The five homologous LhrC sRNAs display exceptionally high sequence and structural similarity, despite being encoded at two distinct genomic loci in *L. monocytogenes*. LhrC1-4 are encoded from the intergenic region between *cysK* and *sul*, while *lhrC5* is found between *lmo0946* and *lmo0947*. Expression of LhrC1–5 is strongly induced under various stress conditions, but not during stationary phase (Christiansen et al., 2006). These sRNAs play crucial roles in virulence, cefuroxime resistance, and heme tolerance (Krawczyk-Balska et al., 2021). Recently, two additional sRNAs—Rli22 and Rli33-1—were identified as novel members of the LhrC family (Mollerup et al., 2016). Although they share structural and functional similarities with LhrC1-5, neither Rli22 nor Rli33-1 interacts with Hfq, distinguishing them from their LhrC counterparts. To date, six target genes have been identified for the LhrC sRNAs, five of which encode virulence-associated surface proteins in *L. monocytogenes*: the adhesin LapB, oligopeptide-binding protein OppA, T-cell activating antigen TcsA, and heme-binding proteins Hbp1 and Hbp2 (Krawczyk-Balska et al., 2021). Structurally, all LhrC family members share a conserved architecture consisting of two stem-loops (designated stem-loop A and the terminator loop) connected by a single-stranded stretch, with multiple CU-rich motifs located in its single-stranded regions.

The LhrC-dependent downregulation mechanism involves base pairing between CU-rich motifs in the sRNAs and AG-rich sequences in the 5′-untranslated region (5′-UTR) of target mRNAs. In most cases, LhrC binding to AG-rich sites overlapping the RBS blocks translation initiation. However, regulation of *tcsA* represents an exception: here, the CU-rich motifs interact with a distal AG-rich site upstream of the RBS, leading to mRNA degradation (Ross et al., 2019). Notably, all characterized LhrC targets show evidence of functional redundancy among the LhrC family members. Furthermore, unlike many bacterial sRNAs, these regulatory interactions occur independently of the Hfq chaperone (Sievers et al., 2014, 2015; Dos Santos et al., 2018; Ross et al., 2019). As anisotropy measurements indicate that *L. monocytogenes* Hfq does not bind at its distal face AAN motifs responsible for preferential binding of RNA by *E. coli* Hfq (Zheng et al., 2016), it has been proposed that this inefficient AAN motif binding, combined with poor recognition of A/U-rich mRNA sequences at the rim face, prevents Hfq from facilitating LhrC sRNA-mRNA annealing (Jørgensen et al., 2020). However, the binding properties of the distal face in *L. monocytogenes* Hfq remain uncertain, as it shares high sequence homology not with *E. coli* Hfq but with *B. subtilis* Hfq, which exhibits distinct target specificity (Someya et al., 2012). Thus, the proposed deficiency of *L. monocytogenes* Hfq in mediating LhrC–mRNA base pairing requires further investigation.

Our recent characterization of the SOS-interfering factor Sif revealed that *lhrC5* constitutes the terminal gene of the ferritin operon, which comprises *fri*, *lmo0944*, *lmo0945*, *sif*, and *lhrC5* (Ładziak et al., 2024). The operon’s first gene encodes ferritin (Fri), an iron-storage protein crucial for iron homeostasis, stress adaptation and virulence in *L. monocytogenes* (Olsen et al., 2005; Krawczyk-Balska et al., 2012; Milecka et al., 2015). The *L. monocytogenes* ferritin, a Dps-family protein, safeguards the cell by oxidizing and storing cytosolic Fe(II) to avert Fenton reaction-mediated toxicity, while concurrently storing the insoluble Fe(III) as internal ferric hydroxide micelles (Haikarainen and Papageorgiou, 2010). The transcriptional regulation of *fri* has been well characterized, with expression driven by three distinct promoters: one σB-dependent and two σA-dependent (Olsen et al., 2005). Furthermore, *fri* transcription is repressed by Fur (ferric uptake regulator) and PerR (peroxide stress regulator) (Rea et al., 2005; Fiorini et al., 2008). However, the post-transcriptional regulation of *fri* and other operon genes remains unexplored.

In this study, we investigated how the sRNA LhrC5 and Hfq post-transcriptionally regulate ferritin expression in *L. monocytogenes* using a combined *in vitro* and *in vivo* approach. We provide the first experimental evidence that Hfq can directly facilitate the interaction between an LhrC-family sRNA and its target mRNA *in vitro*. Importantly, we demonstrate that LhrC5 specifically targets the σA1-derived *fri* mRNA for degradation under osmotic stress *in vivo*, thereby fine-tuning its expression. However, Hfq is not required for LhrC5-mediated *fri* decay under the tested stress conditions. Nevertheless, overexpression of Hfq under conditions of a σA1-to-σB shift of the *fri* promoter (induced by the TetR system) leads to increased degradation of both the σA1- and σB-derived *fri* mRNAs, demonstrating that elevated Hfq levels can downregulate *fri* expression. Taken together, these results highlight the roles of LhrC5 and Hfq in the post-transcriptional control of ferritin expression in *L. monocytogenes*.

## Materials and methods

### Bacterial strains construction and growth conditions

The wild-type strain *L. monocytogenes* EGD-e was used in this study. The isogenic mutant derivatives ΔlhrC1-4 and lhrC5* were constructed in previous work (Ładziak et al., 2024). For the construction of an in-frame deletion mutant of *hfq L. monocytogenes*, the temperature-sensitive shuttle vector pMAD was used (Arnaud et al., 2004). On the template of EGD-e chromosomal DNA, the *hfq* 5′ flanking fragment was amplified using the primers DLhfqFA and DLhfqRB, and the 3′ fragment using the primers DLhfqFC and DLhfqRD. The amplified 5′ and 3′ fragments were spliced by overlap extension PCR, and the obtained product was subsequently cloned into pMAD. The pMAD derivative was used for genes replacement which was performed by double-crossover homologous recombination as described previously (Arnaud et al., 2004). For construction of the strain expressing C-terminal 3xFLAG-tagged Hfq from the *L. monocytogenes* EGD-e chromosome, the temperature-sensitive shuttle vector pAUL-A was used (Schäferkordt and Chakraborty, 1995). Primers Hfq-3xFLAG-1, −2, −3, and −4 were used for a 2-step PCR amplification of a DNA fragment containing the 3xFLAG-tag inserted just before the stop codon of the *hfq* gene. First, two PCR reactions were run with primers Hfq-3xFLAG-1 and −2, or Hfq-3xFLAG-3 and −4, respectively, using chromosomal DNA of *L. monocytogenes* EGD-e as template. Second, the two PCR-fragements were joined by overlap extension PCR and the resulting DNA fragment was inserted into pAUL-A. The resulting plasmid was introduced into *L. monocytogenes* EGD-e and homologous recombination was achieved as described previously (Christiansen et al., 2004).

For the construction of the of the strains expressing translational fusion of σA1 *fri* promoter and *gfp* reporter gene, site-specific listerial integrative vector pIMK3 was used (Monk et al., 2008). DNA fragment comprising the σA1 *fri* promoter was amplified on the template of EGD-e chromosomal DNA using the primers PA1friFA and PA1friRB. DNA fragments comprising *gfp* gene were amplified on the template of pSGFPS1 vector (Rodriguez et al., 2017) using the primers gfpFC and gfpRD. The amplified fragments were spliced by overlap extension PCR, and cloned into pIMK3. The obtained plasmid construct was introduced into *L. monocytogenes* strains. For the construction of the strains overexpressing Hfq in tetR dependent manner, anhydrotetracycline-inducible vector pRMC2 was used (Corrigan and Foster, 2009). DNA fragment comprising the *hfq* gene was amplified on the template of EGD-e chromosomal DNA using the primers HfqF_pRMC2 and HfqR_pRMC2. The amplified fragment was cloned into pRMC2 and introduced into *L. monocytogenes* strains.

All primers used in this study are listed in Supplementary Table S1. *L. monocytogenes* was routinely grown in brain heart infusion broth (BHI, Oxoid) at 37 °C with shaking. When appropriate, cultures were supplemented with kanamycin (50 μg/mL), chloramphenicol (7.5 μg/mL), erythromycin (5 μg/mL) or X-gal (50 mg/mL). For nutrient deficiency stress *L. monocytogenes* was grown in KRM medium, prepared essentially as described previously (Newton et al., 2005). Briefly, KRM medium was based of RPMI 1640 (Sigma), with the following supplements (Sigma): casamino acids (0.1%), adenine (10 μg/mL), tryptophan (50 μg/mL), riboflavin (0.5 μg/mL), biotin (1 μg/mL), thiamine (1 μg/mL), lipoic acid (0.005 μg/mL), (NH4)_6_Mo_7_O_24_·4H_2_O (0.3 μM), CoCl_2_·6H_2_O (3 μM), HBO_3_ (40 μM), CuSO_4_·5H_2_O (1 μM), MnCl_2_ (8 μM), ZnCl_2_ (1 μM). *E. coli* strain Top10 (Invitrogen) was used in cloning experiments, and *E. coli* BL21*hfq*^−^ strain carrying pTyb11/*hfq* (Christiansen et al., 2006) was used for purification of the Hfq of *L. monocytogenes*. *E. coli* strains were grown on Luria-Bertani medium. The LB medium was supplemented with ampicillin (50 μg/mL or 100 μg/mL) or kanamycin (50 μg/mL) when required.

### Green fluorescence assay

Overnight cultures of *L. monocytogenes*, including EGD-e, Δhfq, and lhrC5* carrying the PσA1*fri*-*gfp* translational fusion integrated into the chromosome at the *tRNA* locus, as well as the control strain (wild-type EGD-e carrying the empty pIMK3 vector integrated at the same locus), were grown in BHI medium supplemented with kanamycin at 37 °C with aeration. To obtain cultures for stress experiments, the overnight cultures were diluted 1:100 in fresh BHI medium without antibiotic and grown at 37 °C to an optical density at 600 nm (OD_600_) of 0.35. At this point, the cultures were exposed to the following concentrations of stress factors: 4 μg/mL cefuroxime, 0.09 μg/mL penicillin G, 4% NaCl, 2% ethanol, 2 mM EDTA, and 8 μM hemin. For nutrient deficiency stress, the cultures were collected by centrifugation (5 min, 6,000 × g), washed in saline, and resuspended in KRM medium or KRM medium supplemented with 8 μM hemin. Subsequently, the cultures were grown further at 37 °C for 1 h.

For measurement of *gfp* expression, cells from the *L. monocytogenes* cultures were collected by centrifugation (5 min, 6,000 × g) and resuspended in saline. Next, 200 μL of the cell suspensions were transferred to wells of Corning^®^ 96 Well Black Polystyrene Microplates (black, clear bottom, Merck). Absorbance at 600 nm and GFP fluorescence (excitation 485 nm; emission 528 nm) were measured using a TECAN Infinite^®^ 200 PRO microplate reader.

Raw fluorescence values were corrected by blank subtraction. Background fluorescence was measured from the wild-type strain carrying the empty pIMK3 vector under each experimental condition, using three biological replicates. For all tested strains, the blank-corrected fluorescence values were adjusted to the optical density at 600 nm (OD₆₀₀) by dividing by the corresponding OD₆₀₀ value. The average background signal for each condition was then calculated from the OD₆₀₀-adjusted values of the empty-vector control strain. Finally, the fluorescence values of the reporter strains (pIMK3-PσA1*fri-gfp*) were normalized by dividing by the corresponding average background signal measured under the same growth condition. The results are expressed as relative fluorescence (fold change over background).

### Coimmunoprecipitation and northern blot analysis

The *L. monocytogenes* EGD-e and EGD-e Hfq-3xFLAG strains were grown in BHI medium overnight with shaking at 37 °C. The following morning, each culture was diluted 1:100 into fresh BHI broth and incubated with shaking at 37 °C to the optical density at 600 nm (OD_600_) 0.35. At this point, the culture of each strain was supplemented with 0.09 μg/mL penicillin G, and cultures were grown further at 37 °C. After 60 min of growth, 100 mL of each culture was collected by centrifugation (3 min, 8,000 × g, 4 °C) and snap-frozen in liquid nitrogen. Frozen cells were washed two times in ice-cold CoIP buffer (100 mM Tris-Cl, 1 mM MgCl_2_, 200 mM KCl, pH 7.5), resuspended in 2 mL CoIP buffer supplemented with complete protease inhibitor cocktail, and disrupted using Fastprep. Lysates were centrifuged (5 min, 20,000 × g, 4 °C), and supernatants were transferred to tubes containing washed anti-FLAG M2 magnetic beads. They were subsequently incubated for 2 h at 5 °C with rotation. After that time, anti-FLAG M2 magnetic beads were washed three times with 1 mL CoIP buffer supplemented with a complete protease inhibitor cocktail and then subjected to RNA isolation by phenol/chloroform extraction. The concentration of isolated RNA was determined with a NanoDrop ND-1000 spectrophotometer. 250 ng of isolated RNA in a loading buffer containing 50% formamide and 20% formaldehyde was separated on a formaldehyde agarose gel and subsequently transferred to a Zeta probe nylon membrane (Bio-Rad) by capillarity blotting. For RNA detection, the membranes were pre-hybridized for 1 h in PerfectHyb buffer (Sigma-Aldrich) and then hybridized overnight at 64 °C with ^32^P-labeled double-stranded DNA probes specific for each gene within the operon. The probes were generated using [*α*-^32^P]dATP and the Megaprime DNA labeling system (Amersham Biosciences), following the manufacturer’s instructions. The primers used for probe preparation are listed in Supplementary Table S1. RNA bands were visualized by phosphorimaging using a Typhoon scanner (GE Healthcare). When required, the membranes were stripped by washing in boiling 2 × SSC buffer containing 0.1% SDS at 80 °C with rotation for 1 h. Following confirmation of efficient probe removal, the membranes were re-hybridized with subsequent probes. Band intensities were quantified using ImageQuant software (GE Healthcare).

### EMSA RNA

The Hfq of *L. monocytogenes* for EMSA experiments was purified as described previously (Christiansen et al., 2006), and the EMSA analysis was performed as described previously (Lillebæk and Kallipolitis, 2018). Briefly, the templates for *in vitro* transcription of *fri* mRNA TSS1_σA2, *fri* mRNA TSS2_σB, and *fri* mRNA TSS3_σA1 were prepared on *L. monocytogenes* EGD-e chromosomal DNA while the templates for LhrC5, LhrC5_mut and LhrC4 were produced by PCR using overlapping primers (Supplementary Table S1). RNAs obtained in *in vitro* transcription were purified and, when required, de-phosphorylated and radiolabeled with [*γ*- 32P] ATP. In EMSA experiments, 0.04 pmol of radiolabeled *fri* mRNA and 10 μg yeast tRNA were mixed with an excess of unlabeled sRNA in the presence or absence of the Hfq protein in a total volume of 10 μL for 1 h at 37 °C. Subsequently, samples were separated on a 5% non-denaturing gel at 4 °C. Dried gels were exposed overnight to PhosphorImager screens for imaging. RNA bands were visualized using a Typhoon scanner (GE Healthcare) and band intensities were quantified using ImageQuant software (GE Healthcare).

### Structure probing

5′-end labeled transcript of *fri* mRNA TSS3_σA1, nonlabeled LhrC5 or LhrC5_mut and the Hfq protein were prepared as described for the EMSA experiments. The enzymatic probing was carried out essentially as previously described (Ross et al., 2019). Briefly, 0.1 pmol radiolabeled mRNA and 10 μg yeast tRNA were mixed with 25 pmol of nonlabeled LhrC5 or LhrC5_mut and/or 250 pmol of the Hfq protein, and structure probing RNA interactions were incubated at 37 °C for 1 h before treating the samples with the cleaving agents. For enzymatic digestion, the samples were treated with 2 pg. of RNase A (Invitrogen) and 0.1 U of RNase T1 (Invitrogen) for 2 min at 37 °C. At the same time, for chemical probing, 1 μL 25 mM lead (II) acetate was added to the samples and reactions were incubated for 5 min at 37 °C. Control samples were prepared likewise (except for the cleaving agents) and incubated at 37 °C for the duration of the experiment. For the alkaline hydrolysis ladder, 0.1 pmol of labeled RNA and 10 μg yeast tRNA were mixed with alkaline hydrolysis buffer (Invitrogen) and incubated at 95 °C for 5 min, while for the T1 sequencing ladder, 0.1 pmol of labeled RNA and 10 μg yeast tRNA was denatured and incubated with 0.01 U of T1 RNase (Invitrogen) at 37 °C for 5 min. Samples were separated on an 8% denaturing polyacrylamide gel. RNA bands were visualized by phosphor imaging using a Typhoon scanner (GE Healthcare).

### *In vitro* translation

*In vitro* translation was performed essentially as described previously (Ross et al., 2019). Briefly, translation reactions were prepared using the PURExpress *In Vitro* Protein Synthesis kit (New England Biolabs Inc.) in a total volume of 30 μL. Reactions containing 0.2 pmol *in vitro* transcribed full-length *fri* mRNA TSS3_σA1 ± 50 pmol LhrC5 or LhrC5_mut and ± 500 pmol of the Hfq protein were prepared. Then, 10 U RiboLock RNase Inhibitor (Thermo Scientific) and 1 μL [35S]-methionine (>1,000 Ci/mmol, Hartmann Analytical) were added together with solutions A and B according to the manufacturer’s instructions. Reactions were incubated for 4 h at 37 °C and subsequently analyzed by sodium dodecyl sulfate (SDS)—10% polyacrylamide gel electrophoresis (PAGE). Dried gels were exposed overnight to PhosphorImager screens for imaging. Protein bands were visualized using a Typhoon scanner (GE Healthcare) and band intensities were quantified using ImageQuant software (GE Healthcare).

### Hfq overexpression and northern blot analysis

Overnight cultures of *Listeria monocytogenes* EGD-e strains—either wild-type or carrying the empty pRMC2 vector or the pRMC2/*hfq* vector—were grown at 37 °C with aeration in BHI medium with chloramphenicol. Each overnight culture was diluted 1:100 in fresh BHI broth; for the strain carrying pRMC2 empty vector and pRMC2/*hfq*, the medium was supplemented with chloramphenicol. Cultures were grown at 37 °C to an optical density at 600 nm (OD_600_) of 0.2. At this point, anhydrotetracycline (ATc) was added to the pRMC2 culture to a final concentration of 1 μg/mL, and to the pRMC2/*hfq* cultures to a final concentration of 0.2 or 1 μg/mL. After 20 min of further incubation, cells were harvested by centrifugation (5 min, 8,000 × g), resuspended in BHI supplemented with 4% NaCl containing the corresponding ATc concentration (0.2 or 1 μg/mL for the pRMC2/*hfq* strain and 1 μg/mL for the pRMC2 control), and cultured for an additional 30 min at 37 °C.

Bacterial cells were then harvested by centrifugation (12,000 × g for 3 min at 4 °C) and snap-frozen in liquid nitrogen. Frozen cells were disrupted using a FastPrep instrument and total RNA was extracted with 1 mL of TRItidy G™ Reagent (Applichem). RNA concentration and purity were determined using a NanoDrop ND-1000 spectrophotometer, and integrity was confirmed by agarose gel electrophoresis. Northern blot analysis was performed essentially as described previously (Nielsen et al., 2010). Briefly, 7 μg of total RNA in loading buffer containing 95% formamide and 2 mM EDTA was denatured and separated on a 6% denaturing polyacrylamide gel (300 V, 5 h) at 4 °C. RNA was then transferred to a Zeta-Probe nylon membrane (Bio-Rad) by semi-dry electroblotting. Membranes were pre-hybridized for 1 h in PerfectHyb buffer (Sigma-Aldrich) and subsequently hybridized at 42 °C overnight with specific ^32^P-labeled single-stranded DNA probes *fri* (see Supplementary Table S1). Following hybridization, RNA bands were visualized by phosphor imaging using a Typhoon scanner (GE Healthcare). Band intensities were quantified with ImageQuant™ TL software (GE Healthcare). To determine the relative distribution of the three isoforms within a given sample, the total *fri* signal intensity (sum of σA1 + σA2 + σB) was set to 100%, and the abundance of each individual variant was calculated as a percentage of this total.

### RNA stability analysis

The *L. monocytogenes* EGD-e, Δ*hfq*, lhrC5*, and *ΔlhrC1-4* strains were grown in BHI medium overnight with shaking at 37 °C. The following morning, each culture was diluted 1:100 into fresh BHI broth and incubated with shaking at 37 °C to the optical density at 600 nm (OD_600_) 0.35. At this point, cells were harvested by centrifugation (5 min, 6,000 × g), resuspended in BHI supplemented with 4% NaCl and cultured for an additional 30 min at 37 °C.

For stability analysis under Hfq overexpression conditions, *L. monocytogenes* EGD-e strains carrying the empty pRMC2 vector or pRMC2/*hfq* vector were used. Both strains were grown in the presence of 0.2 μg/mL anhydrotetracycline (ATc). Following ATc induction, exponentially growing cells were exposed to osmotic stress for 30 min. RNA stability was analyzed, as described previously (Ross et al., 2019). Rifampicin was added to a final concentration of 10 μg/mL (from a 10 mg/mL stock solution in DMSO) to inhibit transcription. Immediately before rifampicin addition (time 0), an aliquot of the culture was withdrawn, snap-cooled in liquid nitrogen, and harvested by centrifugation (1 min, 12,000 × g, 4 °C). The pellet was frozen in liquid nitrogen and stored at −80 °C. Following rifampicin treatment, additional samples were collected at 2, 4, 8, and 12 min for the wild-type, Δ*hfq*, lhrC5*, and *ΔlhrC1-4* strains, and at 2, 4, 8, and 15 min for strains harboring pRMC2 or pRMC2/*hfq*. Frozen cells were disrupted using a FastPrep instrument and total RNA was extracted with 1 mL of TRItidy G™ Reagent (Applichem). RNA concentration and purity were determined using a NanoDrop ND-1000 spectrophotometer, and integrity was confirmed by agarose gel electrophoresis. Northern blot analysis was performed essentially as described previously (Nielsen et al., 2010). Briefly, 7 μg of total RNA in loading buffer containing 95% formamide and 2 mM EDTA was denatured and separated on a 4.5% denaturing polyacrylamide gel (170 V, 4.5 h) at 4 °C. RNA was then transferred to a Zeta-Probe nylon membrane (Bio-Rad) by semi-dry electroblotting. Membranes were pre-hybridized for 1 h in PerfectHyb buffer (Sigma-Aldrich) and subsequently hybridized at 42 °C overnight with specific ^32^P-labeled single-stranded DNA probes *fri* or 16S rRNA (see Supplementary Table S1). Following hybridization, RNA bands were visualized by phosphor imaging using a Typhoon scanner (GE Healthcare). Band intensities were quantified with ImageQuant™ TL software (GE Healthcare). For the stability analysis, *fri* band intensities were normalized to the corresponding 16S rRNA signals (loading control). Transcript half-life was then calculated by fitting an exponential decay curve to the normalized data.

### *In silico* predictions

Interactions between LhrC sRNAs and *fri* mRNAs were predicted using the IntaRNA software (Mann et al., 2017). Secondary structure predictions of LhrC sRNAs and *fri* mRNAs were obtained using the Mfold web server (Zuker, 2003).

### Statistics

Statistical analysis were performed by an unpaired *t* test (for assays with only 2 groups), or by ANOVA with a Tukey’s multiple comparisons posttest (for comparison of multiple groups with each other). Two-tailed *p*-values were calculated (for assays with only 2 groups), or adjusted *p*-values were calculated (for comparison of multiple groups with each other), and *p* < 0.05 was considered significant. Decay curves were fitted to a one-phase exponential decay model and compared using the extra sum-of-squares F test. Prism 11 (Graph Pad Software) was used for statistical calculations.

## Results

### Hfq binds ferritin operon transcripts *in vivo*

**Figure 1:**
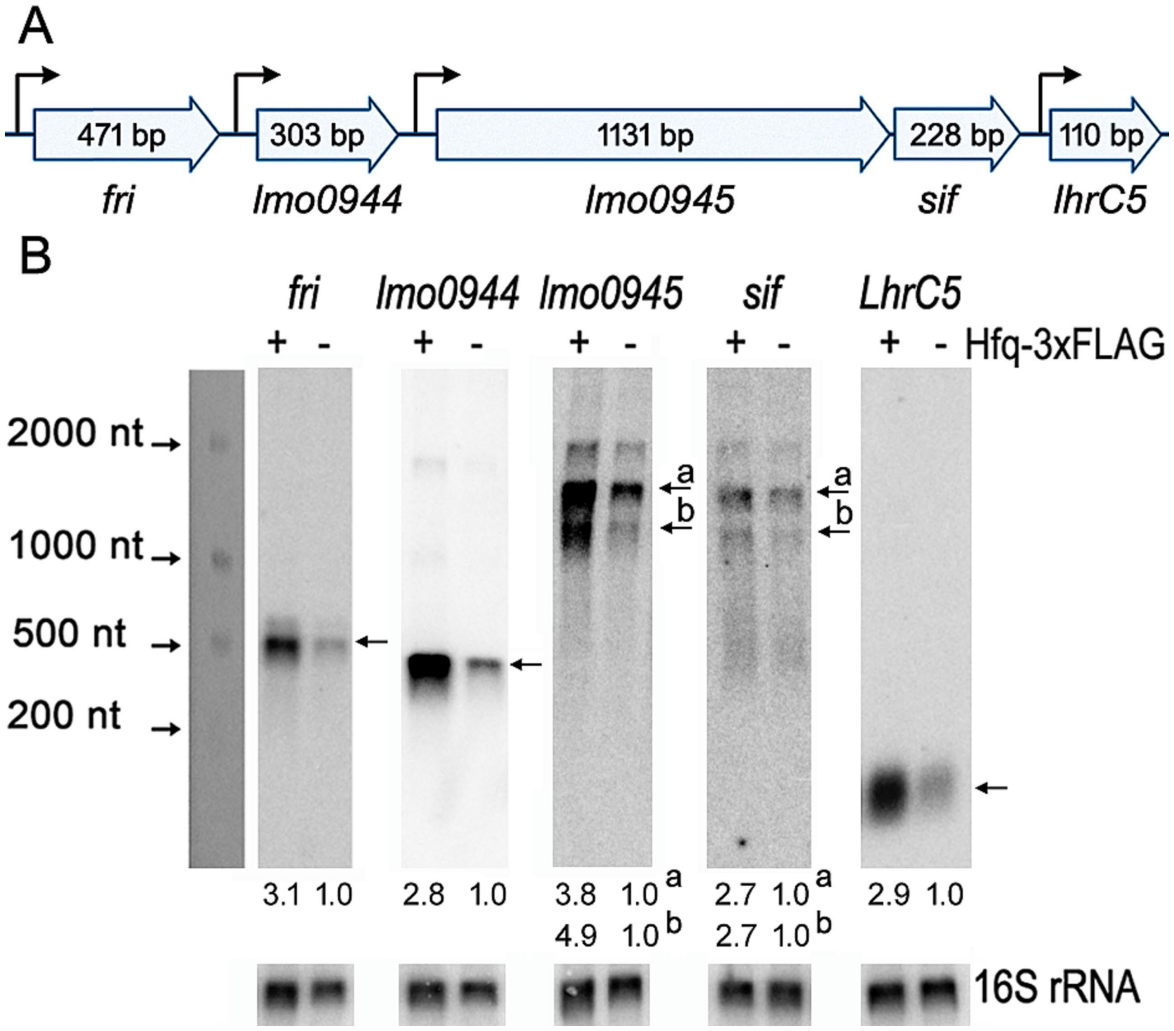
Enrichment of Hfq-bound RNAs from the ferritin operon of *L. monocytogenes*. **(A)** Schematic representation of the ferritin operon genes. Black arrows indicate promoters located within the operon. **(B)** Northern blot analysis of *fri*, *lmo0944*, *lmo0945*, *sif*, and *lhrC5* transcripts co-immunoprecipitated with Hfq from *L. monocytogenes* EGD-e Hfq-3xFLAG (+) and EGD-e (−) using anti-FLAG antibodies. Samples were collected from mid-exponential-phase cultures exposed to penicillin G stress (0.09 μg/mL) for 1 h. The transcripts probed for are indicated at the top; 16S rRNA (bottom) served as a loading control, and a molecular weight marker is shown to the left. Arrows indicate transcripts specifically enriched in *L. monocytogenes* EGD-e Hfq-3xFLAG compared to the EGD-e control. Relative transcript levels (normalized to 16S rRNA) are shown below each lane. The results represent the average of two independent experiments. Statistical significance was determined using an unpaired two-tailed *t*-test, with *p* < 0.05 considered statistically significant.

Our recent study demonstrated that *fri* in *L. monocytogenes* is the first gene of a five-gene operon, which additionally includes *lmo0944*, *lmo0945*, *sif*, and *lhrC5* (Ładziak et al., 2024). The observation that *lhrC5* is the last gene of the ferritin operon led us to hypothesize that LhrC5 and Hfq might be involved in post-transcriptional regulation of the ferritin operon genes. Hfq-dependent regulation typically involves binding of the Hfq protein to target mRNAs. Therefore, to address the potential role of Hfq in controlling the expression of ferritin operon genes, we performed a coimmunoprecipitation (co-IP) assay to detect RNAs specifically associated with Hfq-3xFLAG in *L. monocytogenes*. Hfq-3xFLAG was immunoprecipitated using anti-FLAG antibodies, and the Hfq-bound RNAs were subsequently extracted and detected via northern blot analysis. As a control, we performed a co-IP experiment using the wild-type strain of *L. monocytogenes*. The strains were grown in rich medium (BHI) supplemented with penicillin G to ensure expression of *lhrC5,* which served as a positive control in the co-IP experiments due to the known binding of Hfq to LhrCs *in vivo* (Christiansen et al., 2006). Although transcription of *fri*, *lmo0944*, *lmo0945*, and *lhrC5* is driven by their respective promoters (Figure 1A), multiple transcripts of varying lengths are generated for each gene within the ferritin operon (Ładziak et al., 2024). To account for this transcriptional complexity, we performed northern blot analysis following agarose gel electrophoresis, which effectively separated transcript species ranging from several hundred to several thousand nucleotides in length for subsequent detection. We observed Hfq-3xFLAG-dependent enrichment of several ferritin operon transcripts: a ~ 500 nt monocistronic *fri* mRNA, a ~ 400 nt monocistronic *lmo0944* mRNA, ~1,600 nt and ~1,300 nt polycistronic *lmo0945* and *sif* mRNAs, and the ~100 nt sRNA LhrC5 (Figure 1B). This demonstrates that ferritin operon transcripts interact with Hfq *in vivo*.

### Hfq mediates sRNA-mRNA complex formation *in vitro* between LhrC5 and *fri* mRNA specifically transcribed from the σA1 promoter

Ferritin plays a well-characterized role in stress adaptation and virulence of *L. monocytogenes* (Olsen et al., 2005; Krawczyk-Balska et al., 2012; Milecka et al., 2015). These established functions, combined with our co-IP results, prompted us to investigate the potential involvement of Hfq and LhrC5 in post-transcriptional regulation of *fri* expression.

**Figure 2:**
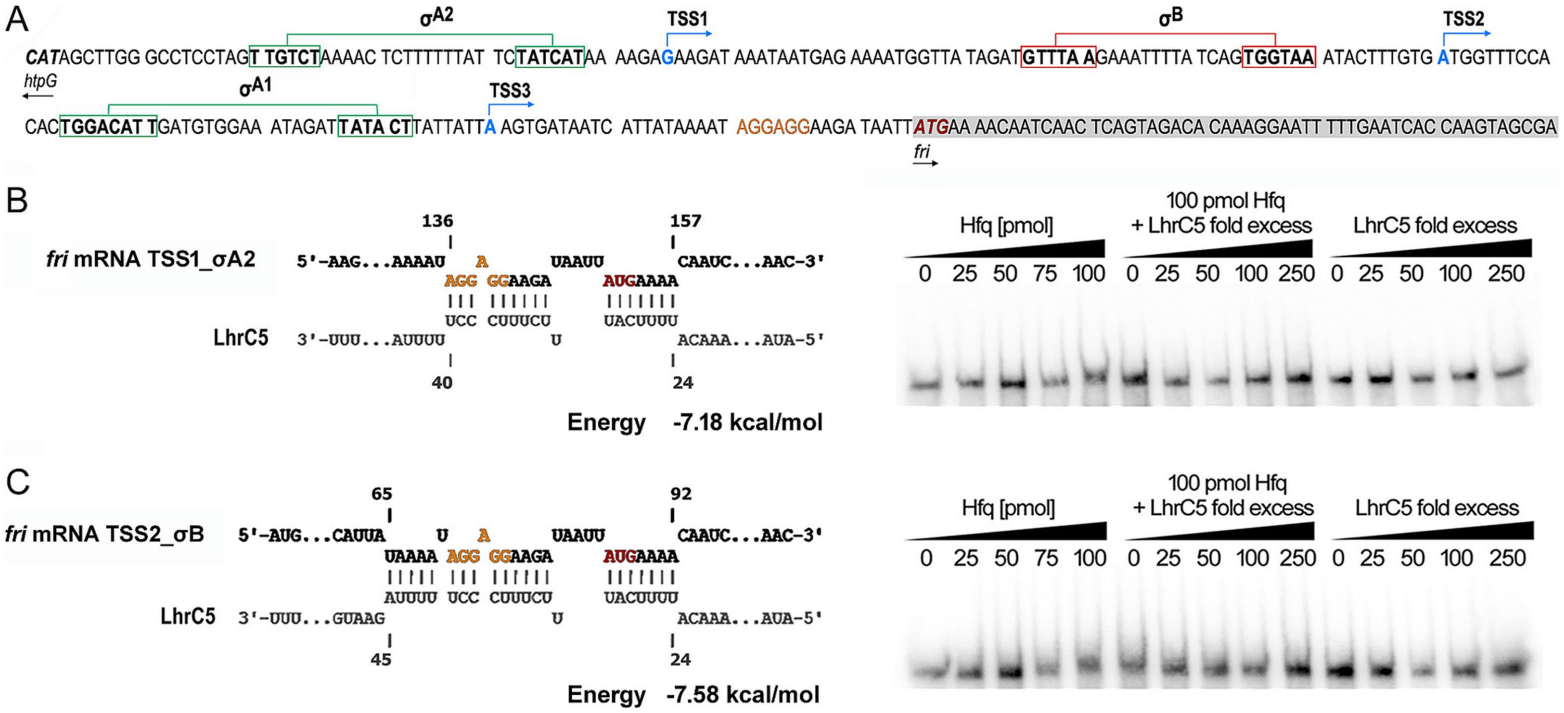
Interactions of LhrC5 and Hfq with *fri* mRNA arising from σ^A2^ and σ^B^ promoters. **(A)** Schematic representation of the promoter region of the *fri* gene. The *fri* sequence is shadowed. The SD region of *fri* is marked in yellow, whereas the start codon is marked in red. Promoter elements corresponding to σ^A1^ and σ^A2^ are marked with green rectangles, while σ^B^ promoter elements are marked with red rectangles. Transcription start sites (TSS1–TSS3) from the individual promoters are marked in blue. **(B)** Analysis of the interaction of LhrC5 with the 5′ end of *fri* mRNAs_TSS1 and involvement of the Hfq chaperone in the formation of sRNA-mRNA complexes. **(C)** Analysis of the interaction of LhrC5 with the 5′ end of *fri* mRNAs_TSS2 and involvement of the Hfq chaperone in the formation of sRNA-mRNA complexes. The results of IntaRNA analysis are shown in the left panel. The SD region of *fri* is marked in yellow, whereas the start codon is marked in red. The predicted free energy of the interactions are indicated. The gel mobility shift assay results are shown in the right panel. 5′-end labeled *fri* mRNAs were shifted with increasing concentrations of Hfq (left) or LhrC5 (right and middle). The LhrC5 binding experiments were performed in the absence (right) or presence (middle) of 100 pmol Hfq. “Fold excess” refers to the amount of LhrC5 added to each sample relative to the amount of labeled *fri* mRNAs. The experiment was performed three times with similar results.

The *fri* gene is transcribed from three distinct promoters—σA2, σB, and σA1—generating monocistronic mRNAs with unique transcription start sites (TSS1, TSS2, and TSS3, respectively) and lengths of 622 nt (mRNA TSS1_σA2), 556 nt (mRNA TSS2_σB), and 507 nt (mRNA TSS3_σA1) (Figure 2A; Olsen et al., 2005).

**Figure 3:**
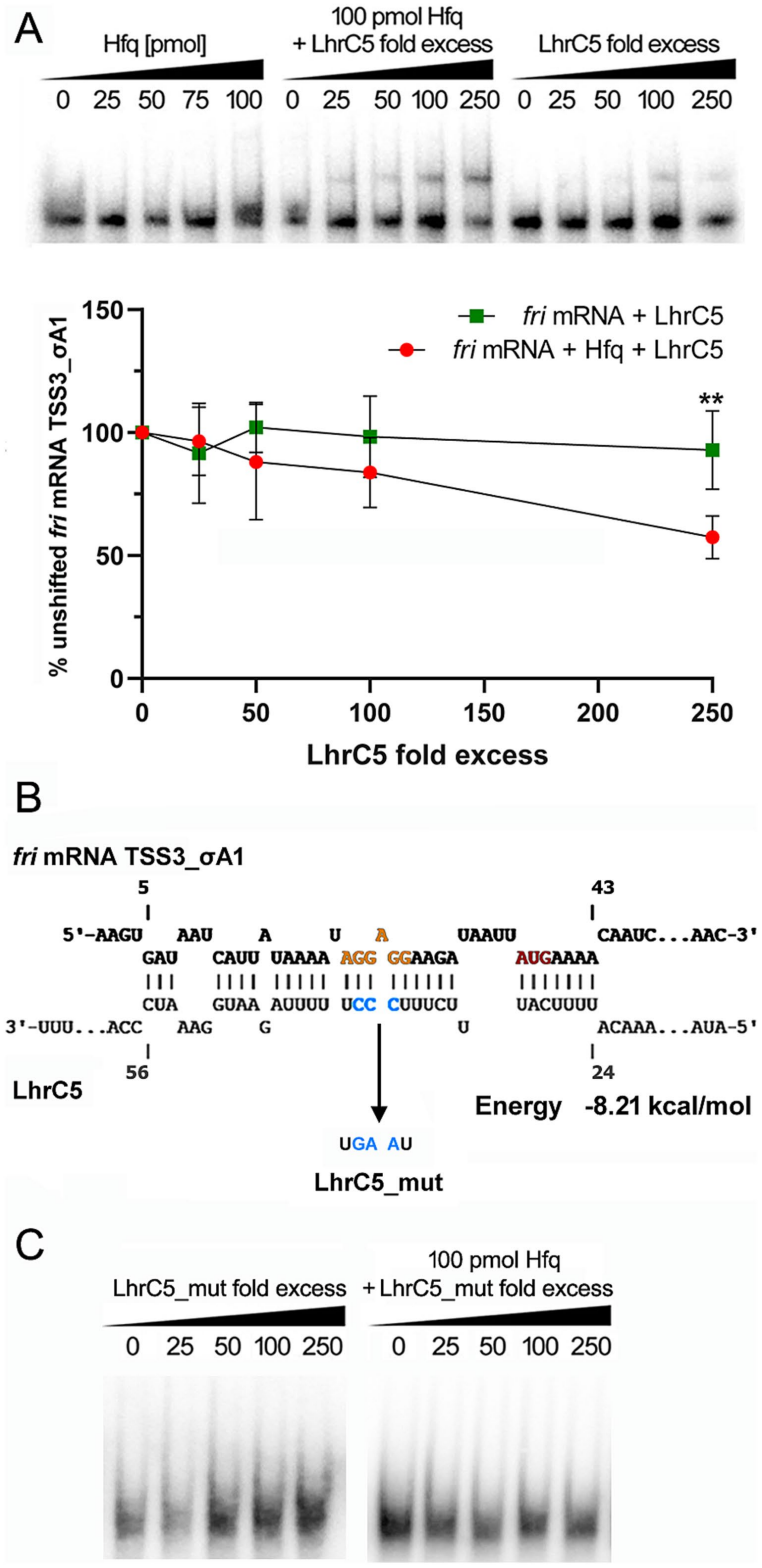
Interactions of LhrC5 and Hfq with *fri* mRNA arising from σ^A1^ promoter. **(A)** The gel mobility shift assay results are shown in the upper panel. 5′-end labeled *fri* mRNA TSS3_σA1 was shifted with increasing concentrations of Hfq (left) or LhrC5 (right and middle). The LhrC5 binding experiments were performed in the absence (right) or presence (middle) of 100 pmol Hfq. “Fold excess” refers to the amount of LhrC5 added to each sample relative to the amount of labeled *fri* mRNA TSS3_σA1. The result of IntaRNA analysis is shown in the lower panel. The SD region of *fri* is marked in yellow, whereas the start codon is marked in red. Quantification of the gel mobility shift assay results is shown in the lower panel. The quantity of *fri* mRNA TSS3_σA1 detected in the absence of LhrC5 and Hfq was set to 100% and used for relative quantification of the level of unbound *fri* mRNA TSS3_σA1 in the presence of LhrC5 alone and in the presence of LhrC5 and Hfq. The values from four experiments are plotted and the error bars indicate standard deviations. Unpaired two-tailed *t* test was used to determine statistical significances. The asterisks indicate significant difference (**p < 0.01). **(B)** IntaRNA-predicted interaction of LhrC5 with the 5′ end of *fri* mRNA_TSS3. The Shine-Dalgarno (SD) sequence and the start codon are highlighted in yellow and red, respectively. The predicted free energy of the interaction (−8.21 kcal/mol) is indicated. Nucleotides of LhrC5 targeted for mutagenesis in loop A are shown in blue; the sequence of the minimal mutant variant (LhrC5_mut) is shown below. **(C)** EMSA analysis of LhrC5_mut binding to *fri* mRNA TSS3_σA1. The 5′-end-labeled *fri* transcript was incubated with increasing concentrations of LhrC5_mut in the absence (right panel) or presence (middle panel) of 100 pmol Hfq. “Fold excess” refers to the molar excess of LhrC5_mut over labeled RNA.

The observed co-IP signal for monocistronic *fri* mRNA could correspond to one or more of its transcript isoforms, as their distinct 5′-UTRs may differentially interact with Hfq and LhrC5. To resolve this, we examined the interactions via *in vitro* binding assays using three 5′-end labeled *fri* mRNA fragments, each containing their respective full 5′-UTR plus 63 nucleotides of the *fri* open reading frame (a 213 nt TSS1-derived fragment, a 148 nt TSS2-derived fragment, and a 99 nt TSS3-derived fragment). To complement the experimental data for LhrC5, we employed the IntaRNA webserver to predict potential binding sites for this sRNA across all *fri* mRNA variants. For *fri* mRNA TSS1_σA2 and mRNA TSS2_σB, IntaRNA analysis predicted potential base pairing between the 5′ end of LhrC5 and the AG-rich Shine-Dalgarno (SD) regions of these transcripts, with predicted interaction energies of −7.18 kcal/mol and −7.58 kcal/mol, respectively (Figures 2B,C, left panels). However, electrophoretic mobility shift assay (EMSA) demonstrated that neither LhrC5 nor Hfq could bind these *fri* mRNA variants (Figures 2B,C, right panels). We next examined LhrC5 binding to *fri* mRNA TSS3_σA1 and the potential role of Hfq in facilitating this interaction. While only weak binding between LhrC5 and the TSS3_σA1 transcript was observed, the addition of Hfq significantly enhanced duplex formation. However, no supershifted band corresponding to a ternary sRNA–mRNA–Hfq complex was detected (Figure 3A), suggesting that Hfq may facilitate the interaction without forming a stable three-component complex. Furthermore, the presence of Hfq alone did not generate a shifted band for this *fri* mRNA variant. Notably, the IntaRNA-predicted interaction between LhrC5 and the TSS3_σA1 transcript is considerably more stable (−8.21 kcal/mol) and exhibits an extended binding interface compared to those with the TSS1_σA2 and TSS2_σB transcripts. Specifically, the 5′ end of LhrC5, encompassing the CCC motif within loop A, is predicted to base-pair with both the Shine-Dalgarno (SD) sequence and the upstream flanking region of TSS3_σA1 (Figure 3B).

To experimentally validate the role of the predicted loop A region in base-pairing with the SD region of *fri* mRNA TSS3_σA1, we introduced mutations in the CCC motif of LhrC5 loop A (Figure 3B). As expected, the mutated LhrC5 variant (LhrC5_mut) failed to bind the wild-type *fri* mRNA TSS3_σA1 (Figure 3C), confirming that loop A is essential for base-pairing with the SD region of *fri* mRNA TSS3_σA1.

The results of the *in vitro* binding experiments indicate that only *fri* mRNA TSS3_σA1 binds efficiently to LhrC5. Given the extended and more stable interaction predicted between LhrC5 and mRNA TSS3_σA1 (ΔG = −8.21 kcal/mol)—but not with mRNA TSS1_σA2 (ΔG = −7.18 kcal/mol) or mRNA TSS2_σB (ΔG = −7.58 kcal/mol)—we analyzed the secondary structures of all three *fri* transcripts used in the *in vitro* binding experiments using the mFold webserver. Interestingly, the region upstream of the SD sequence in mRNA TSS1_σA2 and mRNA TSS2_σB is predicted to form a stem structure (Supplementary Figure S1), which likely prevents this region from participating in extended base pairing with LhrC5 and from binding Hfq.

### Hfq does not stimulate LhrC4—*fri* mRNA TSS3_σA1 complex formation

**Figure 4:**
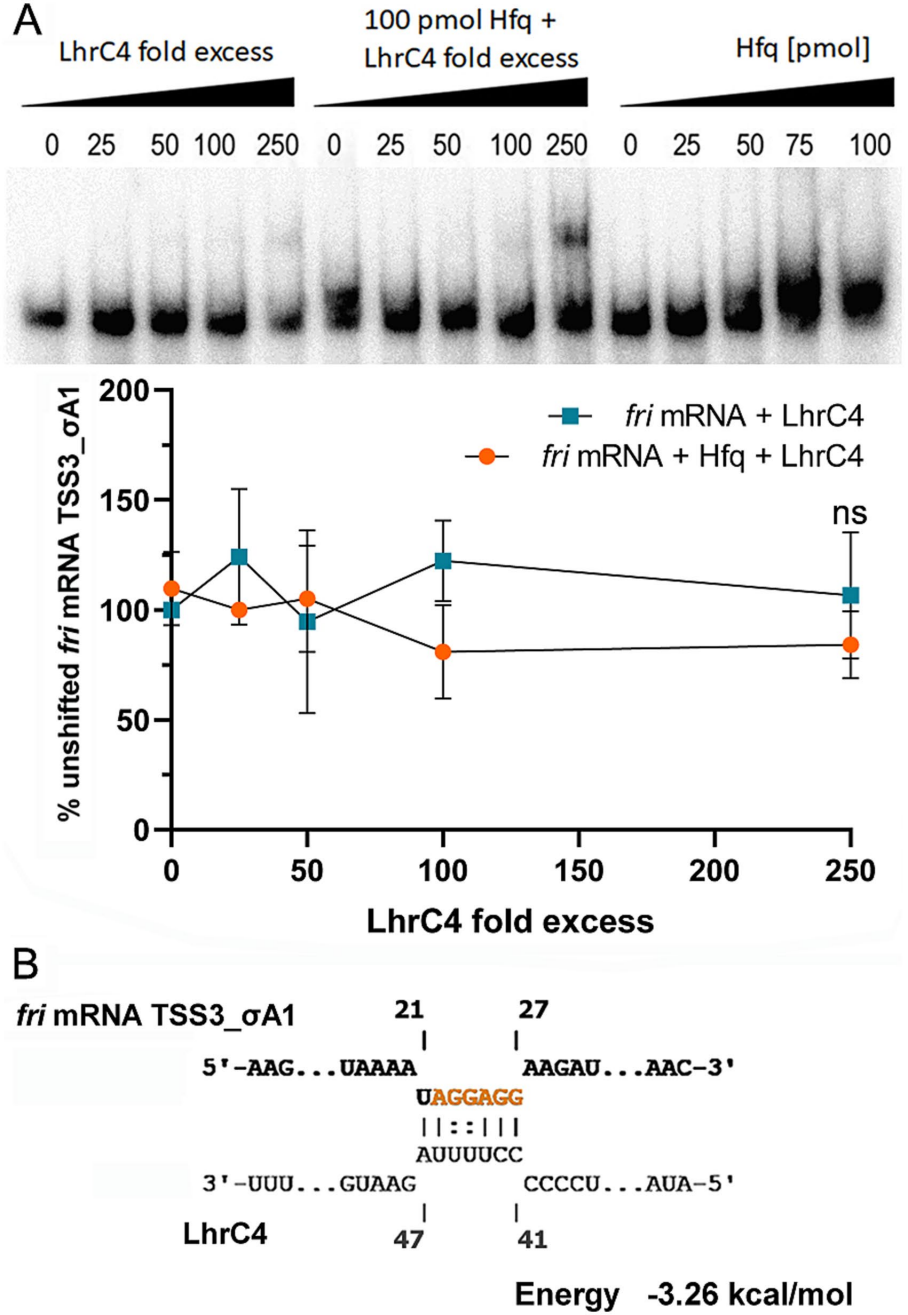
Interactions of LhrC4 and Hfq with *fri* mRNA arising from σ^A1^ promoter. **(A)** The gel mobility shift assay results are shown in the upper panel. 5′-end labeled *fri* mRNA TSS3_σA1 was shifted with increasing concentrations of LhrC4 in the absence (left) or presence (middle) of 100 pmol Hfq, or in the presence of increasing concentrations of Hfq (right). “Fold excess” refers to the amount of LhrC4 added to each sample relative to the amount of labeled *fri* mRNA TSS3_σA1. The result of IntaRNA analysis is shown in the lower panel. The SD region of *fri* is marked in yellow. Quantification of the gel mobility shift assay results is shown in the lower panel. The quantity of *fri* mRNA TSS3_σA1 detected in the absence of LhrC4 and Hfq was set to 100% and used for relative quantification of the level of unbound *fri* mRNA TSS3_σA1 in the presence of LhrC4 alone and in the presence of LhrC4 and Hfq. The values from three experiments are plotted and the error bars indicate standard deviations. Unpaired two-tailed *t* test was used to determine statistical significances; ns, non-significant (*p* > 0.05). **(B)** IntaRNA-predicted interaction of LhrC4 with the 5′ end of *fri* mRNA_TSS3. The Shine-Dalgarno sequence is highlighted in yellow. The predicted free energy of the interaction is indicated.

LhrC5 shares a high degree of sequence and structural similarity with LhrC1–4, and LhrCs are known to interact with Hfq *in vivo* and *in vitro* (Christiansen et al., 2006). Therefore, we investigated whether other members of the LhrC family could also bind *fri* mRNA TSS3_σA1 and whether Hfq plays a role in this interaction. To explore these possibilities, we used the IntaRNA webserver to predict potential LhrC1–4 binding sites in the *fri* TSS3_σA1 transcript. The analysis revealed that all four sRNAs are predicted to bind the AG-rich SD region, with interaction energies substantially lower than that of LhrC5 (−8.21 kcal/mol) (Figure 3B). Specifically, LhrC1 and LhrC4 interact with nucleotides 21–27, with predicted energies of −3.16 kcal/mol and −3.26 kcal/mol, respectively. LhrC3 binds at nucleotides 23–30 with an energy of −4.30 kcal/mol, and LhrC2 interacts with nucleotides 24–30, exhibiting the weakest predicted interaction at −1.15 kcal/mol (see Supplemental Figure S2). Thus, the predicted LhrC1-4—*fri* mRNA TSS3_σA1 complexes all exhibit considerably lower stability compared to LhrC5. For LhrC2 and LhrC3, the interactions involved the CU-rich region of the terminator loop, while for LhrC1 and LhrC4, binding occurred through the CU-rich region of stem-loop A (Supplemental Figure S2). LhrC4 was selected for further analysis to determine whether the CU-rich region of stem-loop A alone is sufficient for sRNA-mRNA complex formation. Gel shift assays revealed weak binding of LhrC4 to the *fri* TSS3_σA1 transcript. Unlike LhrC5, however, the addition of Hfq did not significantly enhance LhrC4-*fri* mRNA TSS3_σA1 complex formation (Figure 4A). The predicted complex involves an interaction between the 5′ end of LhrC4 and a short sequence limited to the SD site of the TSS3_σA1 transcript, with a calculated energy of −3.26 kcal/mol (Figure 4B). This restricted binding interface, reflected in the considerably lower energy compared to LhrC5 (−8.21 kcal/mol), likely accounts for the weaker LhrC4—*fri* mRNA TSS3_σA1 interaction.

### LhrC5 binds to the SD sequence and translation start site of *fri* mRNA TSS3_σA1

**Figure 5:**
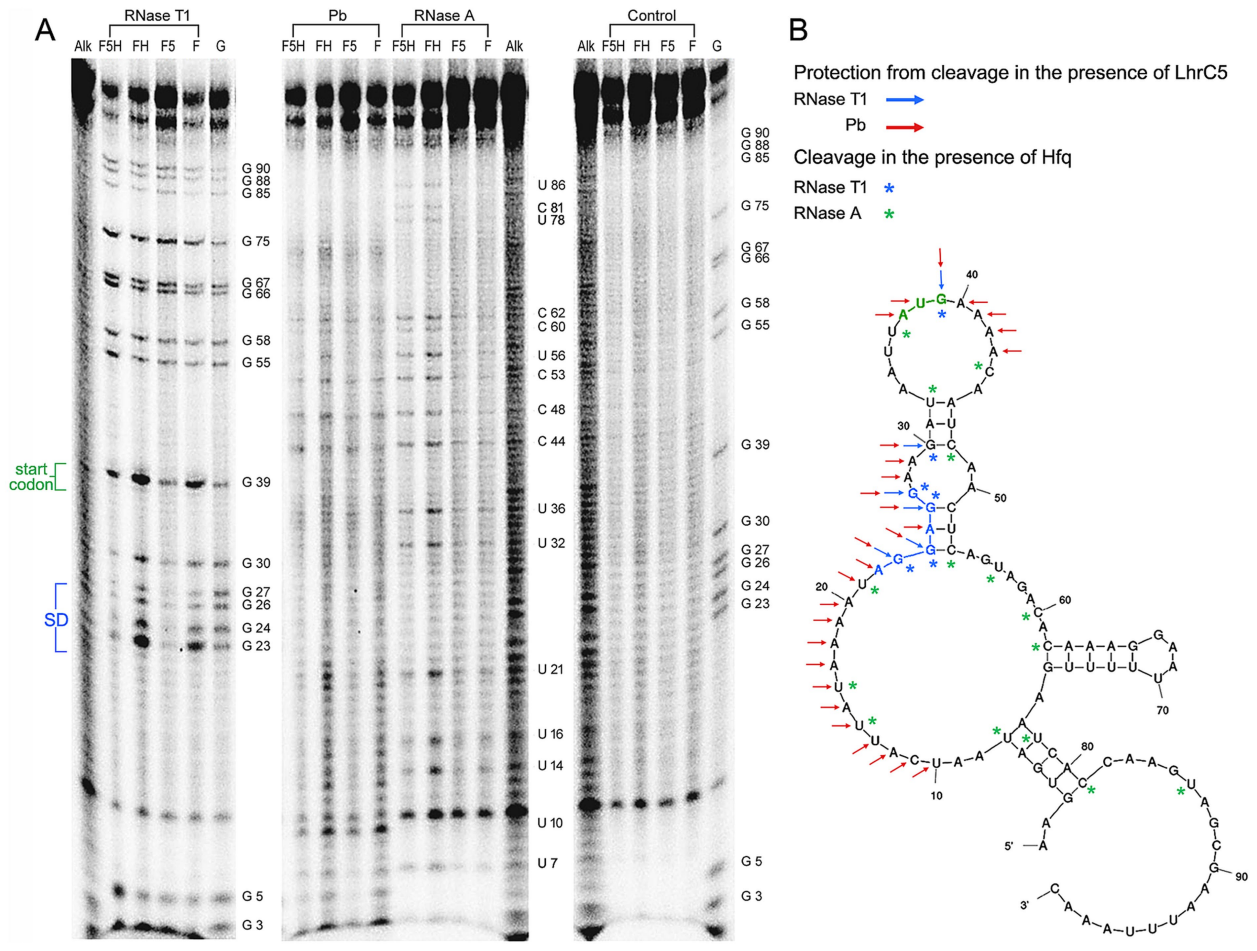
Structure-probing analysis of the interaction between *fri* mRNA TSS3_σA1, LhrC5, and Hfq protein. **(A)** 5′-end labeled *fri* mRNA TSS3_σA1 was partially digested with RNase T1, lead (II) (Pb), or RNase A in the presence of Hfq and non-labeled LhrC5 (F5H), Hfq (FH), non-labeled LhrC5 (F5) or in the absence of other compounds (F). As controls, an alkaline ladder (Alk), RNase T1 ladder (G), and untreated sRNA-mRNA samples (Control) were included. The position of the SD region and the translation start site are marked with blue and green frames, respectively. The experiment was repeated three times with similar results. **(B)** The cleavage pattern of the predicted secondary structure of *fri* mRNA TSS3_σA1. The SD region is marked with blue letters while the translation start site is marked with green letters. The residues protected from cleavage in the presence of LhrC5 (as compared to the cleavage pattern of *fri* mRNA TSS3_σA1 alone) are marked with arrows. The residues exhibiting enhanced cleavage in the presence of Hfq (as compared to the cleavage pattern of *fri* mRNA TSS3_σA1 alone) are marked with asterisks.

**Figure 6:**
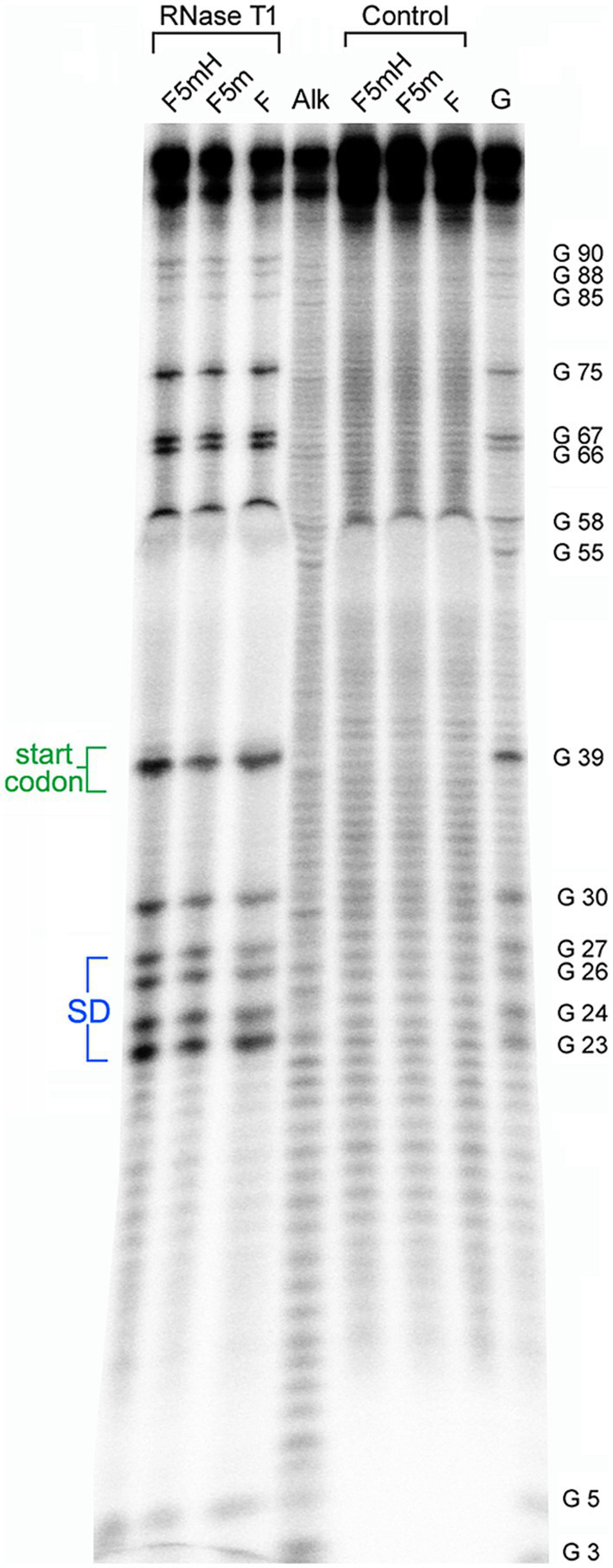
Structure-probing analysis of the interaction between *fri* mRNA TSS3_σA1, LhrC5_mut, and Hfq protein. 5′-end labeled *fri* mRNA TSS3_σA1 was partially digested with RNase T1 in the presence of Hfq and non-labeled LhrC5_mut (F5_mutH), non-labeled LhrC5 (F5_mut), or in the absence of other compounds (F). An alkaline ladder (Alk), an RNase T1 ladder (G), and an untreated RNA sample (Control) were included as controls. The position of the SD region and the translation start site are marked with blue frames, respectively. The experiment was repeated three times with similar results.

To validate the presence of an LhrC5 binding site in the 5′-UTR of *fri* the TSS3_σA1 transcript and further investigate Hfq’s effect on LhrC5–*fri* mRNA TSS3_σA1 complex formation, we performed structure-probing experiments. For these analyses, 5′-end labeled *fri* TSS3_σA1 transcript was treated with RNase T1 (cleaving single-stranded guanosine residues), Lead(II) acetate (targeting single-stranded nucleotides), and RNase A (specific for unpaired cytidine or uridine residues, cleaving 3′ of these bases). We examined sRNA-mRNA interactions in the presence or absence of unlabeled LhrC5 and Hfq. When unlabeled LhrC5 alone was added (F5), we observed clear protection of the AG-rich SD region and translation start site from RNase T1 cleavage (G23 to G39) (Figure 5). Lead(II) acetate probing further revealed that the protected region extended upstream of the SD sequence (U10 to C44) (Figure 5), encompassing both the SD region and translation start site. These findings confirm the predicted binding of LhrC5 to the SD region and translation initiation site of *fri* mRNA TSS3_σA1. When Hfq alone was added (FH), increased cleavage by RNase T1 (G23-G39) and RNase A (U7-U86) was observed (Figure 5), suggesting that the *fri* TSS3_σA1 transcript adopts a slightly more single-stranded conformation in the presence of Hfq. This effect appears to result from Hfq-mediated unwinding of two small double-stranded regions in a hairpin near the RBS of *fri* mRNA TSS3_σA1 (Figure 5). The unwinding of the first double-stranded region was evidenced by enhanced cleavage at G24–G26 within the SD sequence and increased cutting at C53 on the opposite stem strand. Similarly, the second double-stranded region showed enhanced cleavage at G30–U32 and increased cutting at C48 on the complementary strand (Figure 5). Structural probing of the *fri* TSS3_σA1 transcript in the presence of both LhrC5 and Hfq (F5H) resembled the RNase A digestion pattern observed with LhrC5 alone. The SD region and upstream sequence remained protected from RNase T1 (G23-G30) and lead(II) acetate cleavage (U10-U36), with protection levels comparable to those seen with LhrC5 alone (Figure 5). These results indicate that LhrC5 provides comparable protection regardless of Hfq presence, suggesting that Hfq does not enhance LhrC5-*fri* mRNA complex formation under the applied experimental conditions. To confirm that the predicted CCC motif within the loop A region of LhrC5 is responsible for targeting the Shine-Dalgarno (SD) sequence, we performed structure-probing experiments using a mutated LhrC5 variant (LhrC5_mut). As shown in Figure 6, no protection of the AG-rich SD region or the translation start site from RNase T1 cleavage was observed (nucleotides G23–G39). These results confirm that loop A is essential for base-pairing with the SD region of *fri* mRNA TSS3_σA1.

In summary, our findings demonstrate that LhrC5 specifically targets the region spanning both the SD sequence and the translation start codon. Furthermore, Hfq appears to facilitate structural remodeling of the mRNA, thereby potentially increasing the accessibility of sequences involved in LhrC5 base-pairing interactions.

LhrC5 directly represses *fri* translation *in vitro*, and Hfq potentiates this effect, yet neither is detectable in a translational fusion reporter assay *in vivo.*

The *in vitro* binding studies suggested that LhrC5 targets the RBS of *fri* mRNA TSS3_σA1, with Hfq potentially facilitating this interaction. Because direct sRNA binding to an mRNA’s RBS typically represses translation, we employed a reporter gene system to investigate how LhrC5 and Hfq influence *fri* translation *in vivo*. The translational fusion construct, containing the σA1 promoter, the 5′-end of the *fri* coding region (matching the fragment used in *in vitro* studies), and the *gfp* reporter gene, was cloned into the integrative vector pIMK3 (Monk et al., 2008). The recombined vector (pIMK3-*P*σA1*fri*-*gfp*) was subsequently introduced into the wild-type *L. monocytogenes* strain, an ∆*hfq* strain, and a mutant strain lacking *lhrC5.* The empty pIMK3 vector was introduced into the wild-type strain. Both the *fri-gfp* fusion construct and the empty vector were integrated into the chromosome at the *tRNA* locus. A wild-type strain carrying the empty pIMK3 vector served as control. GFP fluorescence was monitored in all strains during cultivation in rich medium (BHI) under both non-stress conditions and stress conditions induced by supplementation with NaCl, ethanol, cefuroxime, EDTA, or hemin. These stressors were selected because they have been previously shown to highly upregulate *lhrC1-5* expression, and some are also known to increase *hfq* expression (Christiansen et al., 2004; Sievers et al., 2014; Dos Santos et al., 2018). Because our co-immunoprecipitation experiments revealed that Hfq binds *fri* mRNA, we also performed fluorescence measurements under penicillin G stress. Additional fluorescence measurements were carried out during growth in minimal medium (KRM) with or without hemin supplementation, since nutrient deprivation has been shown to induce LhrCs expression (Christiansen et al., 2006), and hemin represents the strongest known inducer of LhrCs (Dos Santos et al., 2018).

**Figure 7:**
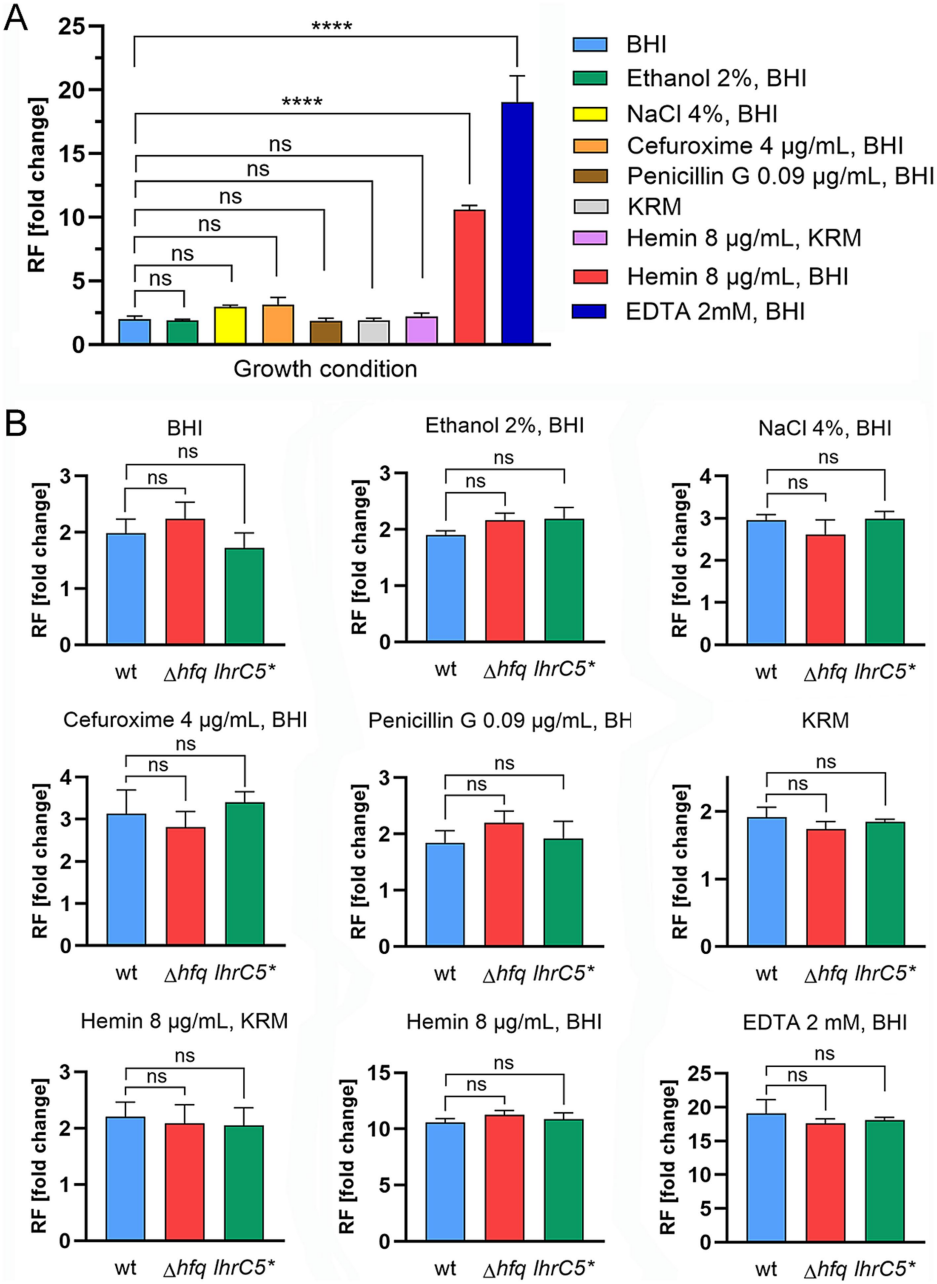
Role of Hfq and LhrC5 in the regulation of *fri* mRNA TSS3_σA1 expression in a reporter assay. **(A)** Expression of *fri* in wild-type under non-stress (BHI) and various stress conditions. **(B)** Expression of *fri* in wild-type, Δ*hfq*, and lhrC5* mutant strains. Green fluorescence was measured in the exponential growth phase for the wild-type *L. monocytogenes* strain EGD-e and its ∆*hfq* or lhrC5* mutant derivatives. All strains carried a *P*σA1*fri-gfp* translational fusion construct integrated into the chromosome at the *tRNA* locus; the empty pIMK3 vector, integrated at the same locus in the wild-type strain, served as a control. Measurements were taken in BHI medium under non-stress conditions and after 1 h of exposure to the following stressors in BHI: 2% ethanol, 4% NaCl, 2 mM EDTA, 4 μg/mL cefuroxime, 8 μM hemin, or 0.09 μg/mL penicillin G. Additionally, strains were analyzed in KRM medium and in KRM medium supplemented with 8 μM hemin. Raw fluorescence values were blank-subtracted and adjusted to OD_600_. Background fluorescence was measured from the empty-vector control (three biological replicates). Reporter fluorescence was normalized by dividing by the corresponding average background signal and is expressed as RF (relative fluorescence; fold change over background). Data are mean ± SD from three biological replicates, each performed in duplicate. ANOVA with a Dunnett’s multiple comparisons posttest was used to determine statistical significances; asterisks indicate significant differences (****p < 0.0001); ns, not significant.

**Figure 8:**
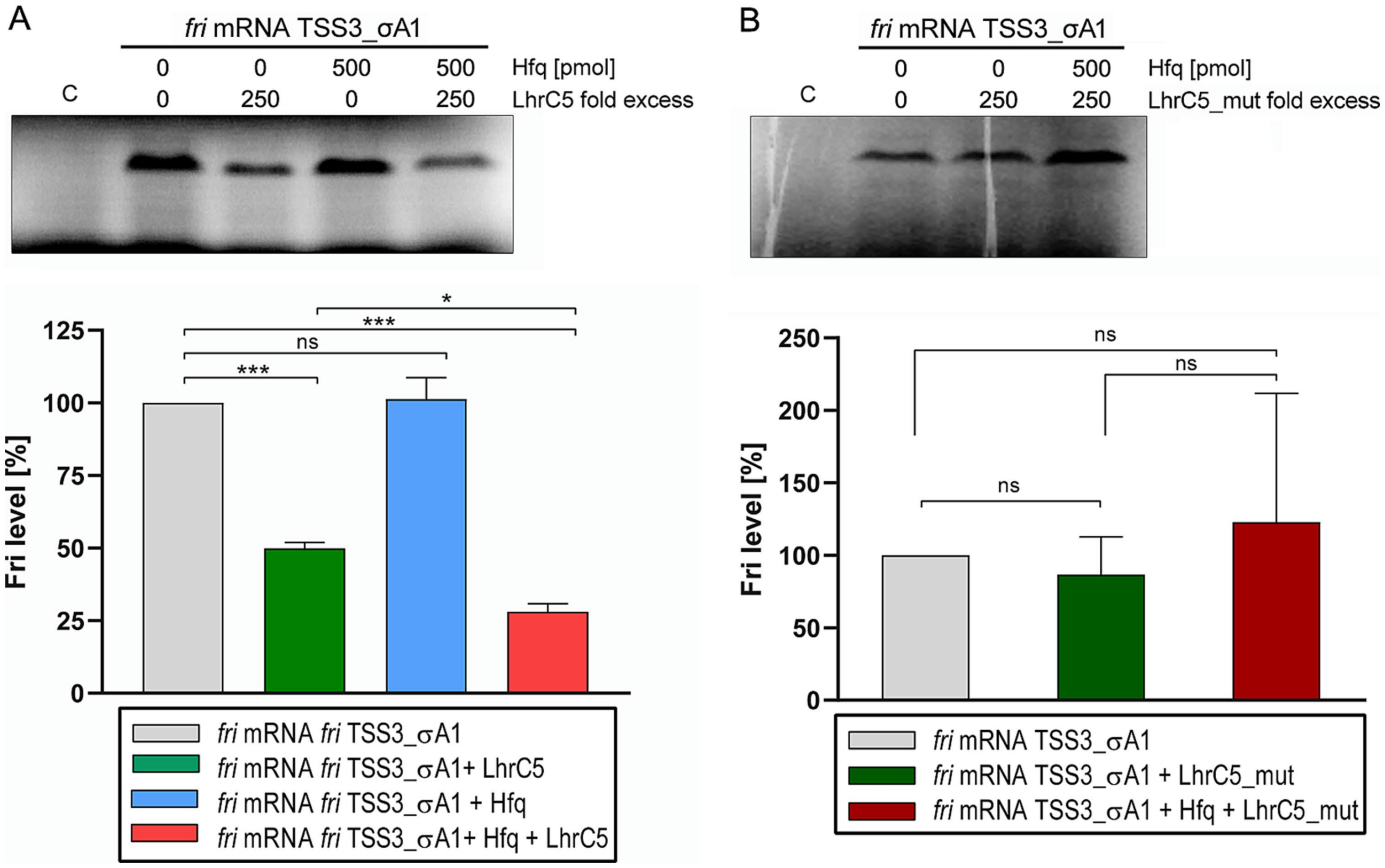
Effect of LhrC5, LhrC5_mut, and Hfq on *in vitro* translation of *fri* mRNA TSS3_σA1. *In vitro*-transcribed *fri* mRNA was used as a template for the synthesis of Fri protein using a reconstituted protein synthesis system. **(A)** *In vitro*-transcribed LhrC5 sRNA and/or 500 pmol of Hfq were added to protein synthesis reactions containing 0.2 pmol *fri* mRNA as a template. The first lane (c) represents a control reaction without template. For quantification of the results (lower panel), the amount of Fri protein produced in the absence of LhrC5 and/or Hfq was set to 100% and used for relative quantification of the level of Fri protein produced in the presence of LhrC5 and/or Hfq. The results shown are the average of two experiments; error bars indicate standard deviations. ANOVA with a Tukey’s multiple comparisons posttest was used to determine statistical significances. The asterisks indicate significant differences (*p < 0.05, ***p < 0.001); ns, non-significant. **(B)** *In vitro*-transcribed LhrC5_mut sRNA was added alone or together with Hfq (500 pmol) to reactions containing 0.2 pmol *fri* mRNA as template, as indicated. The first lane (c) represents the no-template control. Quantification (lower panel) was performed as described in **(A)**.

In fluorescence measurements, the level of *fri* expression under EDTA and hemin stress was significantly higher than under non-stress conditions (growth in BHI), as shown in Figure 7A. This finding is consistent with the previously reported transcriptional derepression of *fri* in response to these stressors (Fiorini et al., 2008; Dos Santos et al., 2018) indicating that the PσA1f*ri-gfp* translational fusion is functional and responds to known stressors. However, no statistically significant differences between wild-type and mutant strains were detected across the experimental conditions (Figure 7B). As further discussed below, the reporter assay likely lacks sensitivity for modest regulatory effects and is included mainly as a complementary, negative observation. Since we did not observe LhrC5- and Hfq-dependent regulation of *fri* translation *in vivo*, we examined their effects on mRNA TSS3_σA1 translation using an *in vitro* system. Specifically, we assessed *fri* translation under four conditions: in the absence of regulators, with LhrC5 alone, with Hfq alone, and with both LhrC5 and Hfq present (Figure 8A). The study revealed that LhrC5 alone reduced Fri protein levels by 2-fold compared to the sRNA-free control. When only Hfq was added to the reaction, Fri production remained unchanged relative to unregulated translation. Notably, the combined presence of Hfq and LhrC5 resulted in a 3-fold reduction of Fri protein, demonstrating that Hfq potentiates LhrC5-mediated repression of *fri* translation (Figure 8A). To confirm that the predicted CCC motif within the loop A region of LhrC5 is responsible for this translational repression, we assessed *fri* translation using a mutated LhrC5 variant (LhrC5_mut). As shown in Figure 8B, the addition of LhrC5_mut did not affect Fri production, which remained comparable to the unregulated translation control.

These results demonstrate that LhrC5 directly represses the translation of *fri* mRNA TSS3_σA1 and that Hfq amplifies this translational repression *in vitro*. Importantly, this regulatory effect observed *in vitro* contrasts with the lack of the regulation seen *in vivo* in the reporter gene assay. We speculate that the discrepancy between the *in vitro* and *in vivo* effects of Hfq and LhrC5 on *fri* translation could result from insufficient cellular levels of Hfq and/or LhrC5 under the stress conditions analyzed. Consequently, the modest regulation observed *in vitro* might fall below the detection limit of the indirect reporter gene assay.

### The *fri* mRNA transcribed from the σA1 is destabilized by LhrC5

**Figure 9:**
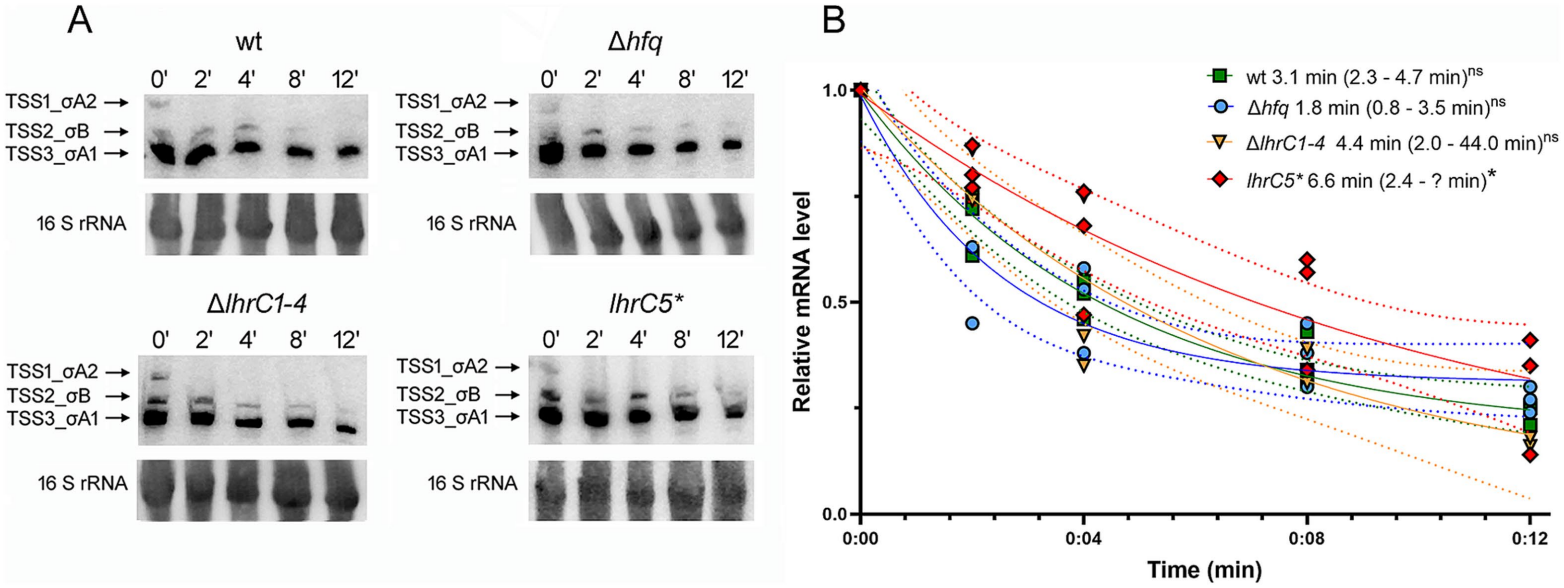
Roles of Hfq, LhrC5, and LhrC1–4 in *fri* mRNA stability. **(A)** Levels of *fri* mRNAs in *L. monocytogenes* EGD-e (WT), Δ*hfq*, lhrC5*, and Δ*lhrC1–4* strains after rifampicin treatment. Exponentially growing cells were exposed to osmotic stress for 30 min. After the addition of rifampicin (time = 0 min), total RNA was extracted from samples harvested at the indicated time points and analyzed by northern blotting. Transcripts derived from the σA2 promoter (TSS1_σA2), the σB promoter (TSS2_σB), and the σA1 promoter (TSS3_σA1) are indicated by arrows; 16S rRNA (bottom) served as a loading control. **(B)** Decay profiles of *fri* mRNA TSS3_σA1 determined from the northern blot data. The quantity of *fri* mRNA at time 0 min was set to 100% and used for the relative quantification of transcript levels at subsequent time points after rifampicin addition. Relative mRNA levels were normalized to 16S rRNA signal intensity. The solid lines represent the nonlinear least squares fit to an exponential decay model; the dotted lines represent the upper and lower limits of the 95% confidence interval for the decay curves. The calculated half-life and 95% confidence interval for each strain are indicated. Data are based on three independent experiments. Statistical significance was determined using the extra sum-of-squares F test; asterisks indicate significant differences (*p < 0.05); ns, not significant.

Inhibition of translation by sRNAs often triggers degradation of the corresponding mRNA. To test this, we directly measured the *in vivo* half-life of *fri* mRNAs to assess the influence of Hfq and LhrC5 on its post-transcriptional regulation. This approach also allowed us to investigate the post-transcriptional regulation of *fri* mRNAs originating from different promoters. Measurements were performed under osmotic stress because this condition induces the expression of both the LhrC1-5 sRNAs and Hfq (Christiansen et al., 2004; Sievers et al., 2014). Furthermore, under these conditions, *fri* transcription is positively regulated by the alternative sigma factor σB (Raengpradub et al., 2008), ensuring the presence of the σB-derived *fri* mRNA in the analyzed samples. Importantly, the transcription start sites of *fri* have been previously mapped (Olsen et al., 2005), allowing the three transcript isoforms to be unambiguously identified based on their known lengths: 622 nt (TSS1_σA2), 556 nt (TSS2_σB), and 507 nt (TSS3_σA1). To determine the involvement of Hfq, LhrC5, and LhrC1-4 in controlling *fri* mRNAs stability in a cellular context, we measured the *in vivo* half-lives of these transcripts in a wild-type *L. monocytogenes*, Δ*hfq*, lhrC5*, and *ΔlhrC1–4* strains. Exponentially growing cells were first exposed to osmotic stress for 30 min to induce LhrC and Hfq expression. Rifampicin was then added at time zero (*t* = 0 min), and samples were collected at specified time points for RNA extraction. We quantified the levels of *fri* mRNAs by northern blot analysis and used the results to calculate the mRNA half-lives and 95% confidence intervals presented in Figure 9B. The half-life of the TSS3_σA1 transcript was slightly but significantly longer in the lhrC5* mutant, showing a 2.1-fold increase compared to the wild-type strain (F-test, *p* = 0.0235). However, due to the substantial biological variability among replicates in the mutant (northern blots from three independent biological replicates and the corresponding decay curves are shown in Supplementary Figure S3), the upper limit of the 95% confidence interval for its half-life could not be reliably determined. Despite this, the significant difference between the decay curves confirms that the transcript is stabilized in the absence of LhrC5. In contrast, the comparable half-lives of mRNA TSS3_σA1 in the wild-type, Δ*hfq*, and Δ*lhrC1–4* strains indicate that neither Hfq nor LhrC1–4 affects its stability. This demonstrates that the TSS3_σA1 transcript is specifically destabilized by LhrC5, but not by LhrC1–4, and that Hfq is not involved in this regulation (Figure 9). Thus, LhrC5 is the only LhrC homolog that regulates mRNA TSS3_σA1 stability, and this regulation is Hfq-independent under the conditions tested. The half-lives of the TSS2_σB and TSS1_σA2 transcripts could not be determined. For TSS1_σA2, the signal rapidly decayed below detectable levels, while for TSS2_σB, the signal intensity showed substantial variation between replicates, in both cases precluding reliable quantification (Figure 9A).

In summary, the half-life experiments demonstrate that among the five LhrC sRNAs, only LhrC5 is capable of destabilizing *fri* mRNA TSS3_σA1, and this regulatory effect represents a fine-tuning modulation rather than strong repression.

However, these stability measurements did not reveal a role for Hfq in mediating LhrC5-driven regulation.

### Both σB- and σA1-derived *fri* mRNA are less stable upon Hfq overexpression

Although osmotic stress induces *hfq* expression (Christiansen et al., 2004), the resulting cellular levels of Hfq may still be insufficient for *fri* regulation. To test this, we investigated whether Hfq overexpression could modulate *fri* expression levels. To this end, the *hfq* gene was cloned downstream of the *tetO* operator sequence in the pRMC2 vector, which provides tight transcriptional control via the TetR repressor and enables fine-tuned, anhydrotetracycline (ATc)-inducible expression of genes of interest (Corrigan and Foster, 2009). First, we tested the effect of Hfq overexpression on the level of *fri* transcripts. The recombinant pRMC2/*hfq* vector and the empty pRMC2 vector were introduced into a wild-type *L. monocytogenes* strain. The strains were then cultured under osmotic stress in the presence of anhydrotetracycline (ATc). The pRMC2/hfq-expressing strain was grown without ATc and with increasing concentrations of ATc (0.2 and 1 μg/mL), whereas the strain carrying the empty pRMC2 vector was grown with ATc (1 μg/mL) to serve as a control for any potential effects of the overexpression system on *fri* mRNA levels. This experimental approach allowed us to specifically monitor changes in *fri* mRNA levels upon Hfq overexpression using the TetR system.

**Figure 10:**
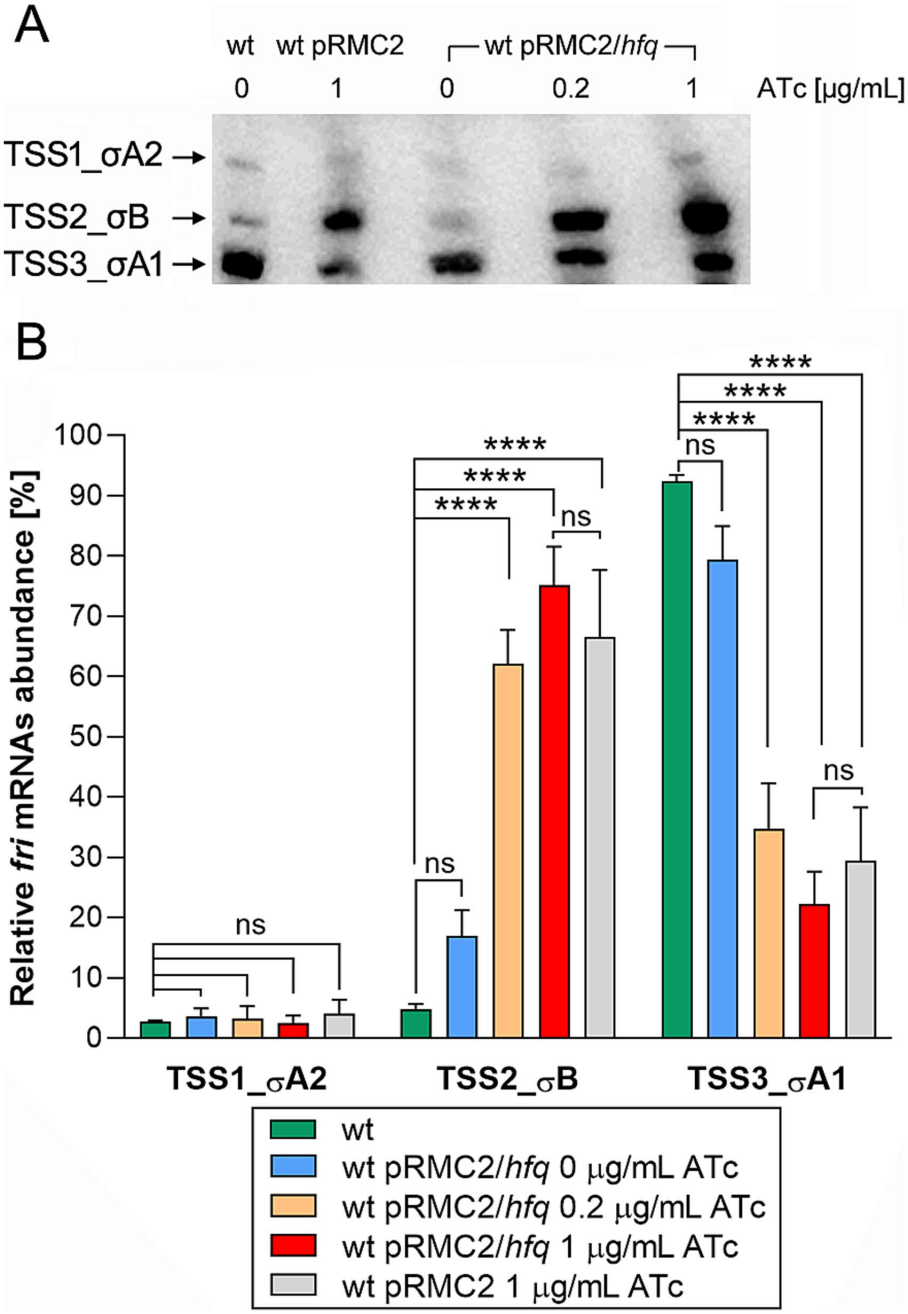
Effect of Hfq overexpression on *fri* mRNA levels. **(A)** Levels of *fri* mRNAs in *L. monocytogenes* EGD-e strains: wild-type (vector-free), wild-type carrying the empty pRMC2 vector (pRMC2), and wild-type carrying the pRMC2/*hfq* vector (pRMC2/*hfq*). Hfq overexpression was induced by the addition of ATc (0.2 or 1 μg/mL) to the pRMC2/*hfq* strain. The wild-type strain and the pRMC2 strain grown with 1 μg/mL ATc served as controls, while the pRMC2/*hfq* strain grown without ATc served as control for basal expression. Total RNA was extracted from exponentially growing cells after a 30-min exposure to osmotic stress and analyzed by northern blotting. Transcripts derived from the σA2 promoter (TSS1_σA2), the σB promoter (TSS2_σB), and the σA1 promoter (TSS3_σA1) are indicated by arrows. **(B)** Relative *fri* mRNA abundance calculated from the northern blot data. To calculate the relative distribution of the three *fri* transcript isoforms (σA1, σA2, and σB) within each sample, the total *fri* mRNA signal intensity for each sample was set to 100%, and the abundance of each individual variant was calculated accordingly. Data represent the mean of three independent experiments; error bars indicate standard deviations. ANOVA with a Tukey’s multiple comparisons posttest was used to determine statistical significances; asterisks indicate significant differences (****p < 0.0001); ns, not significant.

**Figure 11:**
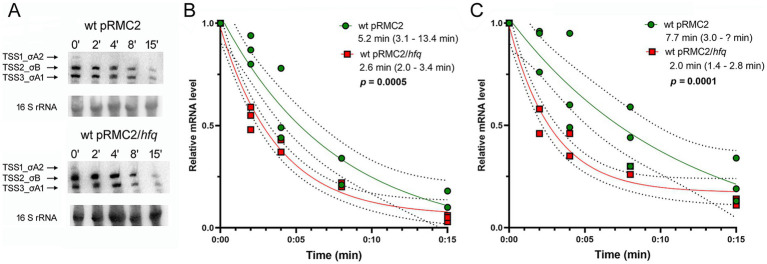
Effect of Hfq overexpression on *fri* mRNA stability. **(A)** Levels of *fri* mRNAs in *L. monocytogenes* EGD-e strains: wild-type carrying the empty pRMC2 vector (pRMC2) and wild-type carrying the pRMC2/*hfq* vector (pRMC2/*hfq*). Following the induction of Hfq overexpression with ATc (0.2 μg/mL), exponentially growing cells were exposed to osmotic stress for 30 min. Rifampicin was then added (time = 0 min), and samples were collected at the indicated time points for RNA extraction. Total RNA was analyzed by northern blotting. Transcripts derived from the σA2 promoter (TSS1_σA2), the σB promoter (TSS2_σB), and the σA1 promoter (TSS3_σA1) are indicated by arrows; 16S rRNA (bottom) served as a loading control. Decay profiles of **(B)** *fri* mRNA TSS2_σB and **(C)** *fri* mRNA TSS2_σA1 determined from the northern blot data. The quantity of *fri* mRNA at time 0 min was set to 100% and used for the relative quantification of transcript levels at subsequent time points after rifampicin addition. Relative mRNA levels were normalized to 16S rRNA signal intensity. The solid lines represent the nonlinear least squares fit to an exponential decay model; the dotted lines represent the upper and lower limits of the 95% confidence interval for the decay curves. The calculated half-life and 95% confidence interval for each strain are indicated, together with the corresponding *p*-value. Data are based on three independent experiments. Statistical significance was determined using the extra sum-of-squares F test.

Under these conditions, the relative abundance of *fri* mRNA TSS1_σA2 remained unchanged across all strains and ATc concentrations tested (Figure 10). In the absence of ATc, the relative abundance of *fri* mRNAs TSS3_σA1 and TSS2_σB was comparable between the wild-type strain and the strain carrying the pRMC2/*hfq* vector. However, upon the addition of 0.2 and 1 μg/mL ATc, the relative abundance of *fri* mRNA TSS3_σA1 in the Hfq-overexpressing strain decreased by 2.3 and 3.6-fold, respectively, compared to the wild-type control. In contrast, under the same conditions, the relative abundance of *fri* mRNA TSS2_σB increased by 3.7 and 4.4-fold, respectively, in the Hfq-overexpressing strain. Notably, the relative abundance of *fri* mRNAs was comparable between the wild-type strain carrying the empty pRMC2 vector and the strain carrying pRMC2/*hfq* when grown in the presence of ATc (Figure 10). Together, these findings indicate that application of the TetR system shifts *fri* expression from the σA1 promoter to the σB promoter, reducing σA1-derived transcripts while increasing those derived from σB. Under these conditions, Hfq overexpression does not significantly influence the overall relative abundance of *fri* mRNAs. To assess whether Hfq regulates *fri* expression post-transcriptionally by affecting mRNA stability, we measured the *in vivo* half-lives of *fri* transcripts under Hfq overexpression conditions. Exponentially growing cells of the strain carrying the empty pRMC2 vector and the strain carrying pRMC2/*hfq* were grown in the presence of 0.2 μg/mL ATc and exposed to osmotic stress for 30 min. Rifampicin was then added (*t* = 0 min), and samples were collected at defined time points for RNA extraction. Levels of TSS3_σA1 and TSS2_σB transcripts were quantified by northern blot analysis and used to calculate their respective half-lives and 95% confidence intervals, which are presented in Figure 11 (northern blots from three independent biological replicates and the corresponding decay curves are shown in Supplemental Figure S4). Compared to the control strain carrying the empty pRMC2 vector, the Hfq-overexpressing strain exhibited a decreased half-life for both transcripts. Specifically, the half-life of *fri* mRNA TSS2_σB was reduced by 2-fold (Figure 11B), whereas the half-life of *fri* mRNA TSS3_σA1 was reduced by 3.9-fold (Figure 11C) in the *L. monocytogenes* pRMC2/*hfq* strain relative to the pRMC2 control. These results indicate that Hfq overexpression leads to reduced stability of *fri* mRNA originating from both the σB- and σA1-dependent promoters.

Collectively, these findings demonstrate that under Hfq-overexpressing conditions, the stability of both *fri* mRNA TSS2_σB and *fri* mRNA TSS3_σA1 is markedly decreased, indicating that Hfq has the potential to post-transcriptionally regulate *fri* expression *in vivo*. However, the observed negative effect on TSS2_σB and TSS3_σA1 may be indirect and requires further investigation.

## Discussion

Recent work has demonstrated that LhrC5, an Hfq-binding sRNA in *L. monocytogenes*, is part of the ferritin operon (Ładziak et al., 2024). This discovery led us to hypothesize that LhrC5—potentially in conjunction with Hfq and other LhrC family members (LhrC1-4)—may post-transcriptionally regulate expression of ferritin operon genes. In support of this hypothesis, co-IP assays demonstrated an association between the mRNAs of ferritin operon genes and Hfq *in vivo*. Given ferritin’s established role in iron homeostasis, stress adaptation and virulence (Olsen et al., 2005; Krawczyk-Balska et al., 2012; Milecka et al., 2015), we specifically investigated the post-transcriptional regulation of *fri* mRNA by Hfq and the LhrC1-5 sRNAs. EMSA analyses of three distinct *fri* mRNA 5′-UTRs revealed that LhrC5 binds weakly to *fri* mRNA TSS3_σA1, and this interaction is significantly enhanced by Hfq. This finding is particularly notable because Hfq has not been observed to facilitate target recognition by LhrC family members (Sievers et al., 2014, 2015; Mollerup et al., 2016; Dos Santos et al., 2018; Ross et al., 2019). Thus, this represents the first documented example of Hfq facilitating the interaction between an LhrC sRNA and its mRNA target *in vitro*. Next, we investigated whether other LhrC sRNAs can also interact with the *fri* mRNA TSS3_σA1 in an Hfq-dependent manner. Given that IntaRNA predictions suggested LhrC4 binds *fri* mRNA TSS3_σA1 with a stability comparable to LhrC1-3 (Supplementary Figure S2), we focused on analyzing complex formation between *fri* mRNA TSS3_σA1 and LhrC4—a well-characterized representative of the LhrC family (Sievers et al., 2014, 2015; Dos Santos et al., 2018; Ross et al., 2019). EMSA analysis showed that LhrC4 only weakly binds the *fri* TSS3_σA1 transcript, and this interaction was not enhanced by Hfq. This finding suggests that among the tested LhrC family members, Hfq specifically facilitates *in vitro* complex formation between the *fri* TSS3_σA1 transcript and LhrC5.

Structure probing analysis demonstrated that LhrC5 binds *fri* mRNA TSS3_σA1 at a region encompassing the SD sequence, upstream elements, and the translation start codon. Notably, previously characterized LhrC-mRNA interactions have been limited to short AG-rich motifs typically overlapping the RBS, with high sequence complementarity being a consistent feature (Sievers et al., 2014, 2015; Dos Santos et al., 2018; Ross et al., 2019). In contrast, the LhrC5–*fri* mRNA TSS3_σA1 interaction exhibits low sequence complementarity and involves a broader mRNA region, not confined to the RBS sequence. Thus, this binding mode differs from the canonical patterns observed for other LhrC targets. The unique ability of LhrC5 to interact with *fri* mRNA TSS3_σA1 likely stems from sequence variations in the regions flanking the conserved CU-rich motifs, which in LhrC1-5 typically target the RBS of mRNAs. These sequence differences appear to enhance LhrC5’s capacity to base-pair with the sequence surrounding the *fri* RBS compared to LhrC1-4 (Supplementary Figure S2). Notably, similar flexibility in target recognition has been observed in multicopy Qrr sRNAs of *Vibrio harveyi* (Shao and Bassler, 2012; Shao et al., 2013), suggesting this may represent a conserved feature of multicopy sRNA systems. The structure probing analysis revealed that Hfq induces a structural rearrangement in *fri* mRNA near the RBS. While Hfq-mediated RNA structural remodeling is a common regulatory mechanism in general, and has also been documented in *L. monocytogenes* (Kavita et al., 2018; Ignatov et al., 2020), the functional significance of this specific conformational change for *fri* regulation remains unclear and warrants further investigation.

In Gram-negative bacteria, a well-established outcome of Hfq-binding sRNAs base-pairing to the RBS of their target mRNAs is translational inhibition (Kavita et al., 2018). This repression is often accompanied by recruitment of the endoribonuclease RNase E to the target mRNAs, leading to degradation that renders the regulation irreversible (Lalaouna et al., 2013). The Hfq-binding sRNA LhrA in *L. monocytogenes* appears to employ a similar mechanism (Nielsen et al., 2010, 2011; Durand et al., 2015). Given this, we assessed the role of Hfq and LhrC5 in *fri* translation. While an inhibitory effect of Hfq and LhrC5 was not observed in an *in vivo* reporter gene assay, we demonstrated that Hfq enhances LhrC5-mediated repression of *fri* TSS3_σA1 translation *in vitro*. Given the modest regulation observed *in vitro*, we hypothesized that the effect might be below the detection limit of the *in vivo* reporter gene assay. This limitation could stem from the high stability of the GFP reporter protein, which constrains its utility in studies of subtle regulation that require rapid reporter turnover to be detected (Li et al., 1998). Alternatively, we speculated that expression of LhrC5 in close vicinity of the *fri* gene would be crucial for down-regulation of the *fri* mRNA TSS3_σA1, ensuring high local concentrations of LhrC5 at the site of regulation.

Based on these considerations and to better capture the postulated regulatory effect, we directly measured the impact of Hfq and LhrC5 on *fri* mRNA stability. The half-life experiments revealed that LhrC5, unlike LhrC1–4 or Hfq, acts as a gentle, post-transcriptional fine-tuner of the *fri* TSS3_σA1 transcript under the conditions tested. Multicopy sRNAs are widespread among bacterial (Waters and Storz, 2009; Göpel and Görke, 2012), where sibling sRNAs may function redundantly (Lenz et al., 2004), additively (Tu and Bassler, 2007), or hierarchically (Urban and Vogel, 2008). For LhrC1-5, previous findings support an additive mode of action as deletion of *lhrC1-4* resulted in growth defects comparable to those observed upon deletion of all five *lhrC* genes in stress tolerance assays (Sievers et al., 2014). Here, we show that LhrC5 specifically regulates the *fri* TSS3_σA1 transcript, providing evidence that beyond an additive function, LhrC sRNAs can also fulfil more specialized regulatory roles. Importantly, the *fri* mRNA stability measurements did not reveal a role for Hfq in mediating LhrC5-driven regulation. The lack of observed Hfq involvement is consistent with previous findings concerning its function in LhrCs-driven regulation; although Hfq in *L. monocytogenes* efficiently binds LhrC1-5, this interaction has not been shown to promote their base-pairing with currently identified target mRNAs nor to affect the stability of these sRNAs (Christiansen et al., 2006; Sievers et al., 2014, 2015). Notably, a similarly ambiguous role of Hfq has been observed in other Gram-positive bacteria. Although Hfq has been shown to interact with *Bacillus subtilis* SR1 and SR2 as well as *Staphylococcus aureus* RNAIII, it neither affects their stability nor facilitates their interactions with target mRNAs (Huntzinger et al., 2005; Geisinger et al., 2006; Heidrich et al., 2006, 2007; Boisset et al., 2007; Preis et al., 2009).

A plausible explanation for our inability to detect a role for Hfq in regulating *fri* mRNAs *in vivo* may be its postulated failure to anneal LhrCs to their mRNA targets, a defect potentially stemming from the inefficient binding properties of the RIM and distal faces of *L. monocytogenes* Hfq (Jørgensen et al., 2020). However, our *in vitro* finding that Hfq facilitates the LhrC5–*fri* mRNA TSS3_σA1 interaction indicates that Hfq may indeed have the capacity to stimulate LhrC5-mRNA complex formation. Another potential factor limiting Hfq’s involvement in LhrC5-driven regulation *in vivo* could be its insufficient cellular concentration under our experimental conditions. In this context, it is worth noting that the inherently low abundance of Hfq in Gram-positive species like *S. aureus* and *B. subtilis* complicates the assessment of whether Hfq variants with impaired RIM-face RNA binding exert regulatory functions *in vivo* (Bohn et al., 2007; Rochat et al., 2015; Christopoulou and Granneman, 2022).

Since Hfq interacts with *fri* transcripts *in vivo* (Figure 1), we sought to determine whether Hfq overexpression could reveal a role for Hfq in the post-transcriptional regulation of *fri.* Application of the TetR system for Hfq overexpression led to a decrease in *fri* mRNA TSS3_σA1 levels and an increase in *fri* mRNA TSS2_σB levels, thereby shifting *fri* expression from the σA1 promoter to the σB promoter, consistent with σB-dependent regulation. Although the TetR system is widely used in various bacterial species, including *L. monocytogenes* (Bertram and Hillen, 2008; Schmitter et al., 2017), to the best of our knowledge, σB response activation has not been described in the context of TetR system application. In *L. monocytogenes*, σB orchestrates the general stress response, governing a regulon of nearly 300 genes (Chaturongakul et al., 2011; Dorey et al., 2019), and expression of *hfq* is σB-dependent (Christiansen et al., 2004). Thus, activation of the σB response appears beneficial in the context of studying Hfq overexpression, as it could be anticipated that the molecular targets of Hfq are expressed alongside Hfq under these conditions.

Subsequent stability studies demonstrated that Hfq overexpression is associated with reduced stability of both *fri* mRNA TSS2_σB and *fri* mRNA TSS3_σA1, consistent with a post-transcriptional effect.

Our observation that Hfq overexpression negatively regulates *fri* expression reveals an additional layer of regulatory complexity. However, the details of this regulation remain to be elucidated, because Hfq, as a global RNA chaperone, undoubtedly reshapes the transcriptome when overexpressed, complicating the distinction between direct and indirect effects.

Future work employing a systems-level approach will be necessary to delineate the complete regulatory circuit. Global transcriptomic analysis under Hfq overexpression conditions should be used to identify co-regulated sRNAs and mRNAs, thereby pinpointing the trans-acting factors responsible for the specific destabilization of σB-derived *fri* mRNAs.

In summary, this study provides mechanistic insight into the post-transcriptional regulation of ferritin in *Listeria monocytogenes*. We identify LhrC5 as a specific regulator that targets the σA1-derived *fri* mRNA for degradation under stress, thereby fine-tuning its expression. Hfq can downregulate *fri* expression when overexpressed, but it is not required for LhrC5-mediated *fri* decay under the tested stress conditions. The observed negative effect of Hfq overexpression on *fri* mRNAs stability may be indirect, possibly mediated through σB activity or an unknown sRNA. Therefore, the molecular basis of the observed Hfq-mediated regulation and its potential interplay with LhrC5 remain to be explored.

We hypothesize that LhrC5-mediated fine-tuning of *fri* expression may be relevant under conditions known to induce both *fri* expression and *lhrC5* transcription, including high osmolarity, oxidative stress, and heme stress. Given that Fri protects against reactive oxygen species, and its expression is induced by iron limitation (Dussurget et al., 2005; Olsen et al., 2005; Lechowicz and Krawczyk-Balska, 2015), we speculate that the LhrC5-mediated destabilization of the σA1-derived *fri* transcript provides a rapid mechanism to fine-tune ferritin levels. This would allow the bacterium to precisely adjust Fri abundance to cellular needs—rather than strongly repressing it—preventing potentially toxic ferritin accumulation when iron is scarce, while maintaining expression levels sufficient for protection against oxidative and other stresses. However, the implications of this regulatory circuit *in vivo* remain to be investigated, and further studies are required to establish its exact role, particularly during infection.

## Data Availability

The original contributions presented in the study are included in the article/Supplementary material, further inquiries can be directed to the corresponding author.

## References

[ref1] Arnaud M. Chastanet A. Débarbouillé M. (2004). New vector for efficient allelic replacement in naturally nontransformable, low-GC-content, gram-positive bacteria. Appl. Environ. Microbiol. 70, 6887–6891. doi: 10.1128/AEM.70.11.6887-6891.2004, 15528558 PMC525206

[ref2] Bertram R. Hillen W. (2008). The application of Tet repressor in prokaryotic gene regulation and expression. Microb. Biotechnol. 1, 2–16. doi: 10.1111/j.1751-7915.2007.00001.x, 21261817 PMC3864427

[ref9020] Boisset S. Geissmann T. Huntzinger E. Fechter P. Bendridi N. Possedko M. . (2007). *Staphylococcus aureus* RNAIII coordinately represses the synthesis of virulence factors and the transcription regulator rot by an antisense mechanism. Genes Dev. 21, 1353–1366. doi: 10.1101/gad.423507 17545468 PMC1877748

[ref9022] Bohn C. Rigoulay C. Bouloc P. (2007). No detectable effect of RNA-binding protein Hfq absence in *Staphylococcus aureus*. BMC Microbiol. 7:10. doi: 10.1186/1471-2180-7-10 17291347 PMC1800855

[ref9003] Chaturongakul S. Raengpradub S. Palmer M. E. Bergholz T. M. Orsi R. H. Hu Y. . (2011). Transcriptomic and phenotypic analyses identify coregulated, overlapping regulons among PrfA, CtsR, HrcA, and the alternative sigma factors sigmaB, sigmaC, sigmaH, and sigmaL in *Listeria monocytogenes*. Appl. Environ. Microbiol. 77, 187–200. doi: 10.1128/AEM.00952-10 21037293 PMC3019704

[ref3] Christiansen J. K. Larsen M. H. Ingmer H. Søgaard-Andersen L. Kallipolitis B. H. (2004). The RNA-binding protein Hfq of *Listeria monocytogenes*: role in stress tolerance and virulence. J. Bacteriol. 186, 3355–3362. doi: 10.1128/JB.186.11.3355-3362.2004, 15150220 PMC415768

[ref4] Christiansen J. K. Nielsen J. S. Ebersbach T. Valentin-Hansen P. Søgaard-Andersen L. Kallipolitis B. H. (2006). Identification of small Hfq-binding RNAs in *Listeria monocytogenes*. RNA 12, 1383–1396. doi: 10.1261/rna.49706, 16682563 PMC1484441

[ref5] Christopoulou N. Granneman S. (2022). The role of RNA-binding proteins in mediating adaptive responses in gram-positive bacteria. FEBS J. 289, 1746–1764. doi: 10.1111/febs.15810, 33690958

[ref6] Corrigan R. M. Foster T. J. (2009). An improved tetracycline-inducible expression vector for *Staphylococcus aureus*. Plasmid 61, 126–129. doi: 10.1016/j.plasmid.2008.10.001, 18996145

[ref9017] Dorey A. Marinho C. Piveteau P. O’byrne C. (2019). Role and regulation of the stress activated sigma factor sigma B (σB) in the saprophytic and host-associated life stages of *Listeria monocytogenes*. Adv. Appl. Microbiol. 106, 1–48. doi: 10.1016/bs.aambs.2018.11.001 30798801

[ref7] Dos Santos P. T. Larsen P. T. Menendez-Gil P. Lillebaek E. M. S. Kallipolitis B. H. (2018). *Listeria monocytogenes* relies on the heme-regulated transporter hrtAB to resist heme toxicity and uses heme as a signal to induce transcription of lmo1634, encoding Listeria adhesion protein. Front. Microbiol. 9:3090. doi: 10.3389/fmicb.2018.03090, 30619169 PMC6305404

[ref9009] Durand S. Tomasini A. Braun F. Condon C. Romby P. (2015). sRNA and mRNA turnover in gram-positive bacteria. FEMS Microbiol. Rev. 39, 316–330. doi: 10.1093/femsre/fuv007 25934118

[ref9013] Dussurget O. Dumas E. Archambaud C. Chafsey I. Chambon C. Hébraud M. . (2005). *Listeria monocytogenes* ferritin protects against multiple stresses and is required for virulence. FEMS Microbiol. Lett. 250, 253–261. doi: 10.1016/j.femsle.2005.07.015 *Erratum in: FEMS Microbiol Lett.* 253:341-2. 16098690

[ref8] Fiorini F. Stefanini S. Valenti P. Chiancone E. De Biase D. (2008). Transcription of the *Listeria monocytogenes* fri gene is growth-phase dependent and is repressed directly by Fur, the ferric uptake regulator. Gene 410, 113–121. doi: 10.1016/j.gene.2007.12.007, 18222616

[ref9] Fuchs M. Lamm-Schmidt V. Lenče T. Sulzer J. Bublitz A. Wackenreuter J. . (2023). A network of small RNAs regulates sporulation initiation in Clostridioides difficile. EMBO J. 42:e112858. doi: 10.15252/embj.2022112858, 37140366 PMC10267692

[ref9018] Geisinger E. Adhikari R. P. Jin R. Ross H. F. Novick R. P. (2006). Inhibition of rot translation by RNAIII, a key feature of *agr* function. Mol. Microbiol. 61, 1038–1048. doi: 10.1111/j.1365-2958.2006.05292.x 16879652

[ref9010] Göpel Y. Görke B. (2012). Rewiring two-component signal transduction with small RNAs. Curr. Opin. Microbiol. 15, 132–139. doi: 10.1016/j.mib.2011.12.001 22197250

[ref10] Haikarainen T. Papageorgiou A. C. (2010). Dps-like proteins: structural and functional insights into a versatile protein family. Cell. Mol. Life Sci. 67, 341–351. doi: 10.1007/s00018-009-0168-2, 19826764 PMC11115558

[ref9011] Heidrich N. Chinali A. Gerth U. Brantl S. (2006). The small untranslated RNA SR1 from the *Bacillus subtilis* genome is involved in the regulation of arginine catabolism. Mol. Microbiol. 62, 520–536. doi: 10.1111/j.1365-2958.2006.05384.x 17020585

[ref9016] Heidrich N. Moll I. Brantl S. (2007). *In vitro* analysis of the interaction between the small RNA SR1 and its primary target *ahrC* mRNA. Nucleic Acids Res. 35, 4331–4346. doi: 10.1093/nar/gkm439 17576690 PMC1935000

[ref9012] Huntzinger E. Boisset S. Saveanu C. Benito Y. Geissmann T. Namane A. . (2005). *Staphylococcus aureus* RNAIII and the endoribonuclease III coordinately regulate *spa* gene expression. EMBO J. 24, 824–835. doi: 10.1038/sj.emboj.7600572 15678100 PMC549626

[ref9002] Ignatov D. Vaitkevicius K. Durand S. Cahoon L. Sandberg S. S. Liu X. . (2020). An mRNA-mRNA interaction couples expression of a virulence factor and its chaperone in *Listeria monocytogenes*. Cell Rep. 30, 4027–4040.e7. doi: 10.1016/j.celrep.2020.03.006 32209466 PMC8722363

[ref11] Jørgensen M. G. Pettersen J. S. Kallipolitis B. H. (2020, 1863). sRNA-mediated control in bacteria: an increasing diversity of regulatory mechanisms. Biochimica et Biophysica Acta:194504. doi: 10.1016/j.bbagrm.2020.194504 32061884

[ref12] Kavita K. de Mets F. Gottesman S. (2018). New aspects of RNA-based regulation by Hfq and its partner sRNAs. Curr. Opin. Microbiol. 42, 53–61. doi: 10.1016/j.mib.2017.10.014, 29125938 PMC10367044

[ref13] Krawczyk-Balska A. Ładziak M. Burmistrz M. Ścibek K. Kallipolitis B. H. (2021). RNA-mediated control in *Listeria monocytogenes*: insights into regulatory mechanisms and roles in metabolism and virulence. Front. Microbiol. 12:622829. doi: 10.3389/fmicb.2021.622829, 33935989 PMC8079631

[ref14] Krawczyk-Balska A. Marchlewicz J. Dudek D. Wasiak K. Samluk A. (2012). Identification of a ferritin-like protein of *Listeria monocytogenes* as a mediator of β-lactam tolerance and innate resistance to cephalosporins. BMC Microbiol. 12:278. doi: 10.1186/1471-2180-12-278, 23176286 PMC3534079

[ref15] Ładziak M. Prochwicz E. Gut K. Gomza P. Jaworska K. Ścibek K. . (2024). Inactivation of lmo0946 (sif) induces the SOS response and MGEs mobilization and silences the general stress response and virulence program in *Listeria monocytogenes*. Front. Microbiol. 14:1324062. doi: 10.3389/fmicb.2023.1324062, 38239729 PMC10794523

[ref16] Lalaouna D. Simoneau-Roy M. Lafontaine D. Massé E. (2013). Regulatory RNAs and target mRNA decay in prokaryotes. Biochimica et Biophysica Acta (BBA)-Gene Regulatory Mechanisms 1829, 742–747. doi: 10.1016/j.bbagrm.2013.02.013 23500183

[ref9006] Lechowicz J. Krawczyk-Balska A. (2015). An update on the transport and metabolism of iron in *Listeria monocytogenes*: the role of proteins involved in pathogenicity. Biometals 28, 587–603. doi: 10.1007/s10534-015-9849-5 25820385 PMC4481299

[ref9001] Lenz D. H. Mok K. C. Lilley B. N. Kulkarni R. V. Wingreen N. S. Bassler B. L. (2004). The small RNA chaperone Hfq and multiple small RNAs control quorum sensing in *Vibrio harveyi* and *Vibrio cholerae*. Cell 118, 69–82. doi: 10.1016/j.cell.2004.06.009 15242645

[ref17] Lillebæk E. M. S. Kallipolitis B. H. (2018). Mutational analysis of sRNA–mRNA base pairing by electrophoretic mobility shift assay. Bacterial Regulatory RNA 1737, 165–176. doi: 10.1007/978-1-4939-7634-8_10, 29484593

[ref18] Link T. M. Valentin-Hansen P. Brennan R. G. (2009). Structure of *Escherichia coli* Hfq bound to polyriboadenylate RNA. Proc. Natl. Acad. Sci. 106, 19292–19297. doi: 10.1073/pnas.0908744106, 19889981 PMC2773200

[ref9014] Li X. Zhao X. Fang Y. Jiang X. Duong T. Fan C. . (1998). Generation of destabilized green fluorescent protein as a transcription reporter. J. Biol. Chem. 273, 34970–34975. doi: 10.1074/jbc.273.52.34970 9857028

[ref19] Mann M. Wright P. R. Backofen R. (2017). IntaRNA 2.0: enhanced and customizable prediction of RNA–RNA interactions. Nucleic Acids Res. 45, W435–W439. doi: 10.1093/nar/gkx279, 28472523 PMC5570192

[ref20] Mikulecky P. J. Kaw M. K. Brescia C. C. Takach J. C. Sledjeski D. D. Feig A. L. (2004). *Escherichia coli* Hfq has distinct interaction surfaces for DsrA, rpoS and poly (a) RNAs. Nat. Struct. Mol. Biol. 11, 1206–1214. doi: 10.1038/nsmb858, 15531892 PMC3071270

[ref21] Milecka D. Samluk A. Wasiak K. Krawczyk-Balska A. (2015). An essential role of a ferritin-like protein in acid stress tolerance of *Listeria monocytogenes*. Arch. Microbiol. 197, 347–351. doi: 10.1007/s00203-014-1053-4, 25352185 PMC4326649

[ref22] Mollerup M. S. Ross J. A. Helfer A.-C. Meistrup K. Romby P. Kallipolitis B. H. (2016). Two novel members of the LhrC family of small RNAs in *Listeria monocytogenes* with overlapping regulatory functions but distinctive expression profiles. RNA Biol. 13, 895–915. doi: 10.1080/15476286.2016.1208332, 27400116 PMC5013991

[ref23] Monk I. R. Gahan C. G. M. Hill C. (2008). Tools for functional postgenomic analysis of *Listeria monocytogenes*. Appl. Environ. Microbiol. 74, 3921–3934. doi: 10.1128/AEM.00314-08, 18441118 PMC2446514

[ref24] Newton S. M. C. Klebba P. E. Raynaud C. Shao Y. Jiang X. Dubail I. . (2005). The svpA-srtB locus of *Listeria monocytogenes*: Fur-mediated iron regulation and effect on virulence. Mol. Microbiol. 55, 927–940. doi: 10.1111/j.1365-2958.2004.04436.x 15661014

[ref25] Nielsen J. S. Larsen M. H. Lillebæk E. M. S. Bergholz T. M. Christiansen M. H. G. Boor K. J. . (2011). A small RNA controls expression of the chitinase ChiA in *Listeria monocytogenes*. PLoS One 6:e19019. doi: 10.1371/journal.pone.0019019, 21533114 PMC3078929

[ref26] Nielsen J. S. Lei L. K. Ebersbach T. Olsen A. S. Klitgaard J. K. Valentin-Hansen P. . (2010). Defining a role for Hfq in gram-positive bacteria: evidence for Hfq-dependent antisense regulation in *Listeria monocytogenes*. Nucleic Acids Res. 38, 907–919. doi: 10.1093/nar/gkp1081, 19942685 PMC2817478

[ref27] Olsen K. N. Larsen M. H. Gahan C. G. M. Kallipolitis B. Wolf X. A. Rea R. . (2005). The Dps-like protein Fri of *Listeria monocytogenes* promotes stress tolerance and intracellular multiplication in macrophage-like cells. Microbiology 151, 925–933. doi: 10.1099/mic.0.27552-0, 15758237

[ref28] Panja S. Schu D. J. Woodson S. A. (2013). Conserved arginines on the rim of Hfq catalyze base pair formation and exchange. Nucleic Acids Res. 41, 7536–7546. doi: 10.1093/nar/gkt521, 23771143 PMC3753642

[ref9007] Preis H. Eckart R. A. Gudipati R. K. Heidrich N. Brantl S. (2009). CodY activates transcription of a small RNA in *Bacillus subtilis*. J. Bacteriol. 191, 5446–5457. doi: 10.1128/JB.00602-09 19542274 PMC2725614

[ref9015] Raengpradub S. Wiedmann M. Boor K. J. (2008). Comparative analysis of the sigma B-dependent stress responses in *listeria monocytogenes* and *Listeria innocua* strains exposed to selected stress conditions. Appl. Environ. Microbiol. 74, 158–171. doi: 10.1128/AEM.00951-07 18024685 PMC2223194

[ref29] Rea R. Hill C. Gahan C. G. M. (2005). *Listeria monocytogenes* PerR mutants display a small-colony phenotype, increased sensitivity to hydrogen peroxide, and significantly reduced murine virulence. Appl. Environ. Microbiol. 71, 8314–8322. doi: 10.1128/AEM.71.12.8314-8322.2005, 16332818 PMC1317367

[ref30] Robinson K. E. Orans J. Kovach A. R. Link T. M. Brennan R. G. (2014). Mapping Hfq-RNA interaction surfaces using tryptophan fluorescence quenching. Nucleic Acids Res. 42, 2736–2749. doi: 10.1093/nar/gkt1171, 24288369 PMC3936774

[ref9008] Rochat T. Delumeau O. Figueroa-Bossi N. Noirot P. Bossi L. Dervyn E. . (2015). Tracking the elusive function of *Bacillus subtilis* Hfq. PLoS One 10:e0124977. doi: 10.1371/journal.pone.0124977 25915524 PMC4410918

[ref31] Rodriguez M. D. Paul Z. Wood C. E. Rice K. C. Triplett E. W. (2017). Construction of stable fluorescent reporter plasmids for use in *Staphylococcus aureus*. Front. Microbiol. 8:2491. doi: 10.3389/fmicb.2017.02491, 29312199 PMC5735104

[ref32] Ross J. A. Thorsing M. Lillebæk E. M. S. Teixeira Dos Santos P. Kallipolitis B. H. (2019). The LhrC sRNAs control expression of T cell-stimulating antigen TcsA in *Listeria monocytogenes* by decreasing tcsA mRNA stability. RNA Biol. 16, 270–281. doi: 10.1080/15476286.2019.1572423, 30706751 PMC6380316

[ref33] Santiago-Frangos A. Woodson S. A. (2018). Hfq chaperone brings speed dating to bacterial sRNA. Wiley Interdiscip. Rev. 9:e1475. doi: 10.1002/wrna.1475, 29633565 PMC6002925

[ref34] Sauer E. Schmidt S. Weichenrieder O. (2012). Small RNA binding to the lateral surface of Hfq hexamers and structural rearrangements upon mRNA target recognition. Proc. Natl. Acad. Sci. 109, 9396–9401. doi: 10.1073/pnas.1202521109, 22645344 PMC3386104

[ref35] Schäferkordt S. Chakraborty T. (1995). Vector plasmid for insertional mutagenesis and directional cloning in Listeria spp. BioTechniques 19, 720–725, 8588903

[ref9004] Shao Y. Bassler B. L. (2012). Quorum-sensing non-coding small RNAs use unique pairing regions to differentially control mRNA targets. Mol. Microbiol. 83, 599–611. doi: 10.1111/j.1365-2958.2011.07959.x 22229925 PMC3262071

[ref9005] Shao Y. Feng L. Rutherford S. T. Papenfort K. Bassler B. L. (2013). Functional determinants of the quorum-sensing non-coding RNAs and their roles in target regulation. EMBO J. 32, 2158–2171. doi: 10.1038/emboj.2013.155 23838640 PMC3730234

[ref36] Schmitter S. Fieseler L. Klumpp J. Bertram R. Loessner M. J. (2017). TetR-dependent gene regulation in intracellular *Listeria monocytogenes* demonstrates the spatiotemporal surface distribution of ActA. Mol. Microbiol. 105, 413–425. doi: 10.1111/mmi.13706, 28508453

[ref37] Schu D. J. Zhang A. Gottesman S. Storz G. (2015). Alternative Hfq-sRNA interaction modes dictate alternative mRNA recognition. EMBO J. 34, 2557–2573. doi: 10.15252/embj.201591569, 26373314 PMC4609186

[ref38] Schumacher M. A. Pearson R. F. Møller T. Valentin-Hansen P. Brennan R. G. (2002). Structures of the pleiotropic translational regulator Hfq and an Hfq–RNA complex: a bacterial Sm-like protein. EMBO J. 21, 3546–3556. doi: 10.1093/emboj/cdf322, 12093755 PMC126077

[ref39] Sievers S. Lund A. Menendez-Gil P. Nielsen A. Storm Mollerup M. Lambert Nielsen S. . (2015). The multicopy sRNA LhrC controls expression of the oligopeptide-binding protein OppA in *Listeria monocytogenes*. RNA Biol. 12, 985–997. doi: 10.1080/15476286.2015.1071011, 26176322 PMC4615310

[ref40] Sievers S. Sternkopf Lillebæk E. M. Jacobsen K. Lund A. Mollerup M. S. Nielsen P. K. . (2014). A multicopy sRNA of *Listeria monocytogenes* regulates expression of the virulence adhesin LapB. Nucleic Acids Res. 42, 9383–9398. doi: 10.1093/nar/gku630, 25034691 PMC4132741

[ref41] Someya T. Baba S. Fujimoto M. Kawai G. Kumasaka T. Nakamura K. (2012). Crystal structure of Hfq from *Bacillus subtilis* in complex with SELEX-derived RNA aptamer: insight into RNA-binding properties of bacterial Hfq. Nucleic Acids Res. 40, 1856–1867. doi: 10.1093/nar/gkr892, 22053080 PMC3287200

[ref9021] Tu K. C. Bassler B. L. (2007). Multiple small RNAs act additively to integrate sensory information and control quorum sensing in *Vibrio harveyi*. Genes Dev. 21, 221–233. doi: 10.1101/gad.1502407 17234887 PMC1770904

[ref9019] Urban J. H. Vogel J. (2008). Two seemingly homologous noncoding RNAs act hierarchically to activate glmS mRNA translation. PLoS Biol. 6:e64. doi: 10.1371/journal.pbio.0060064 18351803 PMC2267818

[ref42] Waters L. S. Storz G. (2009). Regulatory RNAs in bacteria. Cell 136, 615–628. doi: 10.1016/j.cell.2009.01.043, 19239884 PMC3132550

[ref43] Zheng A. Panja S. Woodson S. A. (2016). Arginine patch predicts the RNA annealing activity of Hfq from gram-negative and gram-positive bacteria. J. Mol. Biol. 428, 2259–2264. doi: 10.1016/j.jmb.2016.03.027, 27049793 PMC4884477

[ref44] Zuker M. (2003). Mfold web server for nucleic acid folding and hybridization prediction. Nucleic Acids Res. 31, 3406–3415. doi: 10.1093/nar/gkg595, 12824337 PMC169194

