# Supplementary material for: The sRNA LhrC5 and Hfq post-transcriptionally regulate ferritin expression in Listeria monocytogenes
Source: Front Microbiol. 2026 Jun 26;17:1771046. doi: 10.3389/fmicb.2026.1771046 (PMC13350326; doi:10.3389/fmicb.2026.1771046)
Supplement: Supplementary file 1 [file Data_Sheet_1.PDF]

Title: The sRNA LhrC5 and Hfq post-transcriptionally regulate ferritin expression in *Listeria monocytogenes*

Patrycja Gomza[1], Katarzyna Ścibek[1], Magdalena Ładziak[1], Michał Burmistrz[1], Eva M.S. Lillebaek[2], Birgitte H. Kallipolitis[2], Agata Krawczyk-Balska[1]*

[1] Department of Molecular Microbiology, Biological and Chemical Research Centre, Faculty of Biology, University of Warsaw, Warsaw, Poland

[2] Department of Biochemistry and Molecular Biology, Faculty of Natural Sciences, University of Southern Denmark, Odense, Denmark

SUPPLEMENTARY MATERIALS

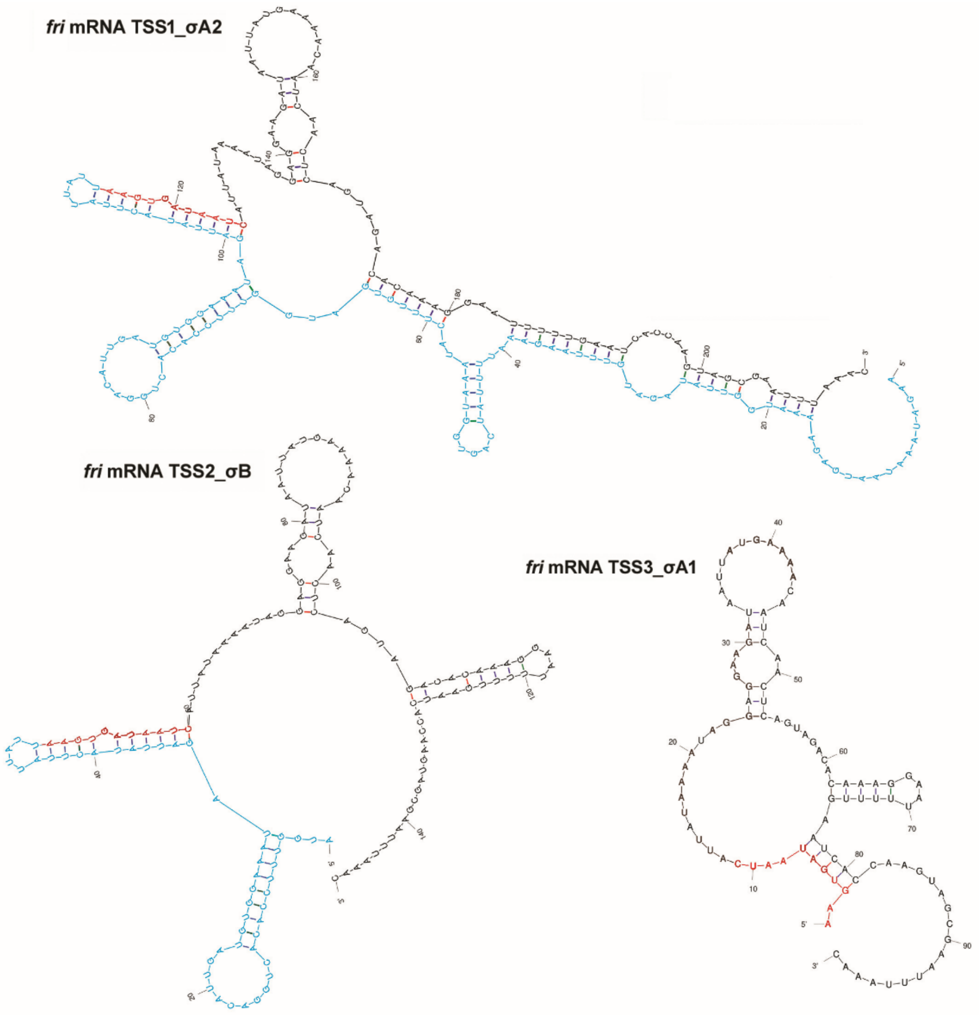

Supplemental Fig S1. Predicted secondary structures of the *fri* mRNA 5′ fragments used in *in vitro* binding experiments: a 213 nt *fri* mRNA 5′ fragment transcribed from the σA2 promoter (*fri* mRNA TSS1_σA2), a 148 nt *fri* mRNA 5′ fragment transcribed from the σB promoter (*fri* mRNA TSS2_σB), and a 99 nt *fri* mRNA 5′ fragment transcribed from the σA1 promoter (*fri* mRNA TSS3_σA1). The 5′ ends of *fri* mRNA TSS1_σA2 and *fri* mRNA TSS2_σB, which are absent in *fri* mRNA TSS3_σA1, are marked in blue. The fragment of region upstream of the SD sequence in *fri* mRNA TSS3_σA1 containing nucleotides predicted to interact with LhrC5, but forming a stem structure in *fri* mRNA TSS1_σA2 and *fri* mRNA TSS2_σB, is highlighted in red.

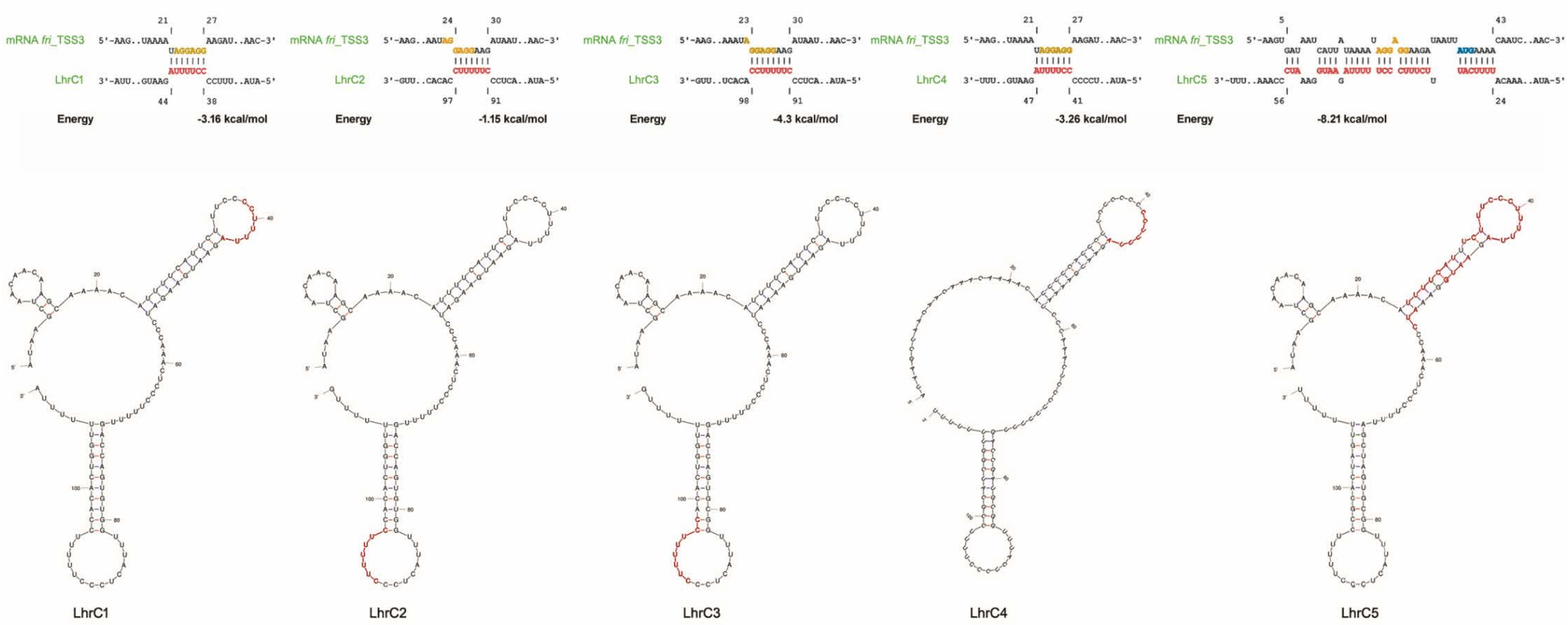

Supplemental Fig S2. Predicted sRNA-mRNA interactions between LhrC1-5 and *fri* mRNA transcribed from σA1 promoter (mRNA *fri*_TSS3). An AG-rich Shine-Dalgarno region of *fri* mRNA predicted to interact with LhrC1-5 is marked in yellow, whereas the start codon of *fri* mRNA predicted to interact with LhrC5 is marked in blue. The predicted regions of interaction in LhrC1-5 are marked in red and involve the CU-rich region of terminator loop in LhrC2 and LhrC3, and CU-rich region of stem-loop A in LhrC1, LhrC4 and LhrC5.

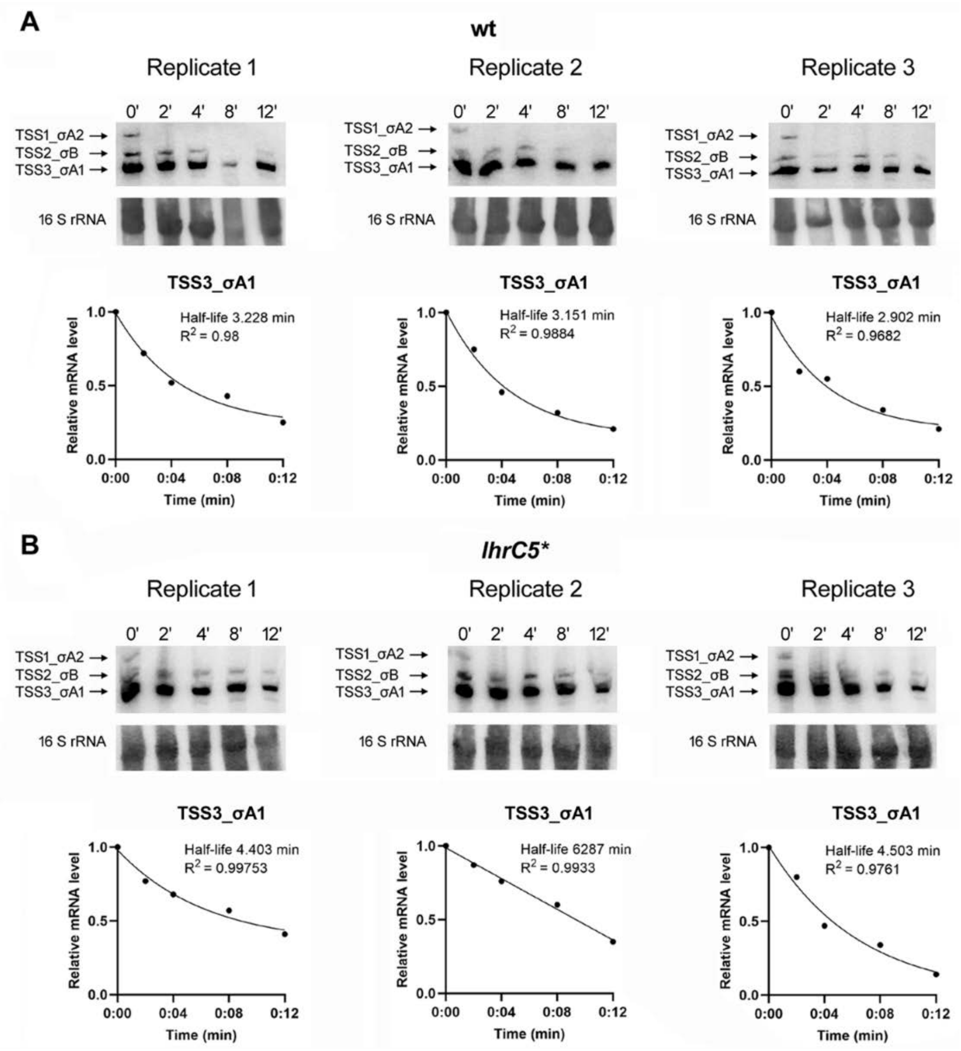

Supplemental Fig S3. Northern blot analysis for determining the stability of *fri* mRNAs in *L. monocytogenes* EGD-e strains: (A) wild-type (wt) and (B) *lhrC5** mutant after rifampicin treatment. Exponentially growing cells were exposed to osmotic stress for 30 minutes. After the addition of rifampicin (time = 0 min), total RNA was extracted from samples harvested at the indicated time points and analyzed by northern blotting. Transcripts derived from the σA2 promoter (TSS1_σA2), the σB promoter (TSS2_σB), and the σA1 promoter (TSS3_σA1) are

indicated by arrows; 16S rRNA (bottom) served as a loading control. Half-lives of *fri* mRNA variants were calculated from the northern blot data. The quantity of *fri* mRNA at time 0 min was set to 100% and used for the relative quantification of transcript levels at subsequent time points after rifampicin addition. Relative mRNA levels were normalized to 16S rRNA signal intensity. The half-life was determined based on an exponential decay curve fitted to the experimental data. Experiments were conducted in biological triplicates.

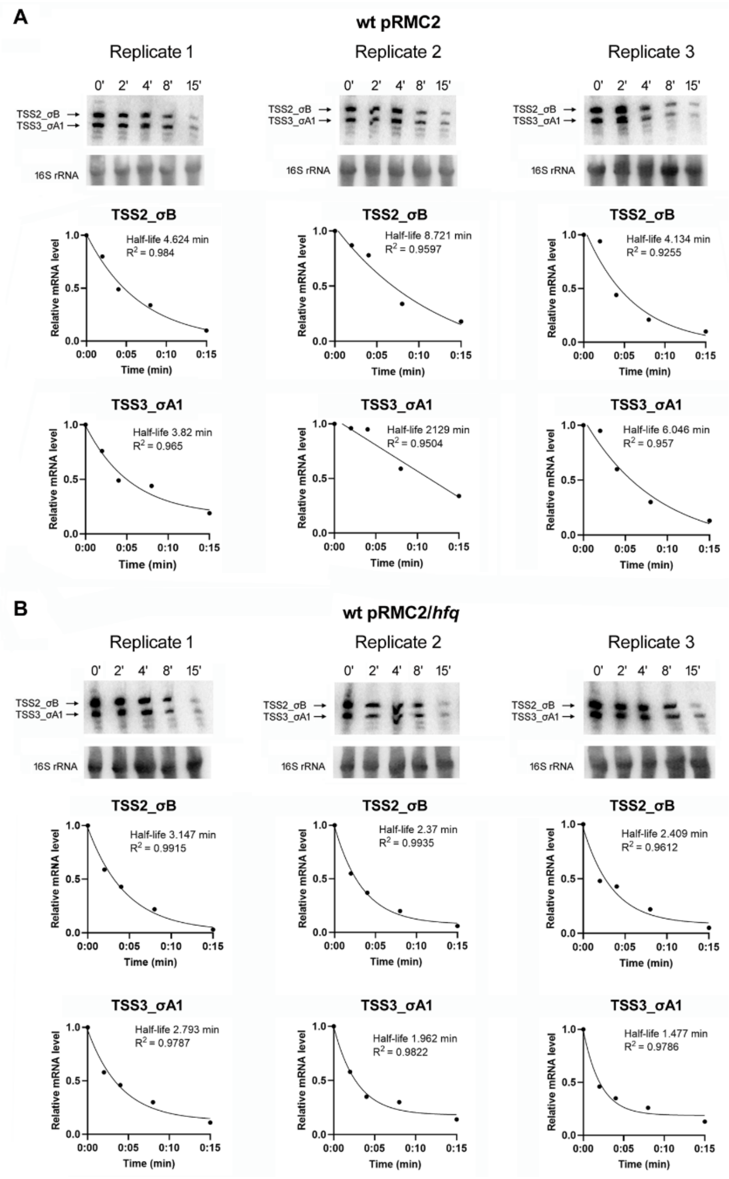

Supplemental Fig S4. Northern blot analysis for determining the stability of *fri* mRNAs in *L. monocytogenes* EGD-e strains: (A) wild-type carrying the empty pRMC2 vector and (B) wild-type carrying the pRMC2/*hfq* vector after rifampicin treatment. Following the induction of Hfq overexpression with ATc (0.2 µg/mL), exponentially growing cells were exposed to osmotic stress for 30 minutes. Rifampicin was then

added (time = 0 min), and samples were collected at the indicated time points for RNA extraction. Total RNA was analyzed by northern blotting. Transcripts derived from the σA2 promoter (TSS1_σA2), the σB promoter (TSS2_σB), and the σA1 promoter (TSS3_σA1) are indicated by arrows; 16S rRNA (bottom) served as a loading control. Half-lives of *fri* mRNA variants were calculated from the northern blot data. The quantity of *fri* mRNA at time 0 min was set to 100% and used for the relative quantification of transcript levels at subsequent time points after rifampicin addition. Relative mRNA levels were normalized to 16S rRNA signal intensity. The half-life was determined based on an exponential decay curve fitted to the experimental data. Experiments were conducted in biological triplicates.

Supplemental Table S1. Primers used in this study.

Name	Sequence (5'→ 3')	Further information
	**Mutants construction**	
**DLhfqFA**	TGGGTCGACGAAGGTTTAGTGACAGAAGCG	Forward primer for upstream flanking region of *hfq*. Restriction enzyme site (underlined). Used for Δ*hfq* construction.
**DLhfqRB**	GCATCAGGCATAATTTCCCTCTCCAATCTC	Reverse primer for upstream flanking region of *hfq*. Used for Δ*hfq* construction.
**DLhfqFC**	**AGGGAAATTATGCCTGATGC**GGAATAAGCAC	Forward primer for downstream flanking region of *hfq*. Anneals with DLhfqRB (bold). Used for Δ*hfq* construction.
**DLhfqRD**	TGGAGATCTCAGCCGAAATATTGCGCAC	Reverse primer for downstream flanking region of *hfq*. Restriction enzyme site (underlined). Used for Δ*hfq* construction.
**Hfq-3xFLAG-1**	GGGGTCTAGAACTACCGAAGCCGAAGCG	Primer for insertion of the 3xFLAG-tag at the C-terminal part of the coding region of *hfq.* XbaI site is underlined.
**Hfq-3xFLAG-2**	**GTCGATATCATGATCTTTATAATCACCGTCATGGTCTTT GTAGTC**TTCCGCATCAGGATTTAAAGC	Primer for insertion of the 3xFLAG-tag (bold) at the C-terminal part of the coding region of *hfq*. Region that anneals with Hfq-3xFLAG-3 is underlined.
**Hfq-3xFLAG-3**	**GATTATAAAGATCATGATATCGACTACAAAGATGACG ACGATAAA**TAAGCACGTTACTTAAAAGAC	Primer for insertion of the 3xFLAG-tag (bold) at the C-terminal part of the coding region of *hfq*. Region that anneals with Hfq-3xFLAG-2 is underlined.

**Hfq-3xFLAG-4**	CCCCGGATCCAGCCGAAATATTGCGCAC	Primer for insertion of the 3xFLAG-tag at the C-terminal part of the coding region of *hfq*. BamHI site is underlined.
	**σA1 *fri* - *gfp* fusions**	
**PA1friFA**	GCGTCGACGTCTCCACACTGGACATTGATGTG	Forvard primer for σA1 promoter of *fri*. AatII restriction enzyme site (underlined). Used for translational fusion of σA1 promoter of *fri* and *gfp* construction.
**PA1friRB**	GTTTAAATTCGCTACTTGGTGATTCA	Reverse primer for σA1 promoter of *fri* Used for translational fusion of σA1 promoter of *fri* and *gfp* construction.
**gfpFC**	**GTAGCGAATTTAAAC**CCCGGGAGCAAAGGAG	Forvard primer for upstream region of *gfp*. Anneals with PA1friRB (bold). Used for translational fusion of σA1 promoter of *fri* and *gfp* construction.
**gfpRD**	TACGGTACCGATATCAAAATTATACATGTCAACGATAAT AC	Reverse primer for downstream flanking region of *gfp*. KpnI restriction enzyme site (underlined). Used for transcriptional and translational fusion of σA1 promoter of *fri* and *gfp* construction.
	***hfq* overexpression**	
**HfqF_pRMC2**	CAGGGTAC**CTCTATCAATGATAGAG**GAGAGGGAA ATTATGAAACAAG	Forvard primer for *hfq*. KpnI restriction enzyme site (underlined), tetO operator sequence (bold). Used for overexpression of *hfq* from TetR promoter.
**HfqR_pRMC2**	CTGAGATCTTCAGACAAGACTAATCGTAGC	Reverse primer for *hfq*. BglII restriction enzyme site (underlined). Used for overexpression of *hfq* from TetR promoter.
	**Northern blot analysis**	
**Fri NB F**	GGCGAACAAATGGATGAAGTA	Forward primer for double stranded probe for *fri* mRNA
**Fri NB R**	CAATACCTTGTTGATATTCGTC	Reverse primer for double stranded probe for *fri* mRNA
**lmo0944 NB F**	GGTAATGGAATTCGACTTTTTGC	Forward primer for double stranded probe for *lmo0944* mRNA
**lmo0944 NB R**	GGTTCATAGTCGATTTTCCAG	Reverse primer for double stranded probe for *lmo0944* mRNA
**lmo0945 NB F**	CCAAGACGCGGCGAAAAAGC	Forward primer for double stranded probe for *lmo0945* mRNA
**lmo0945 NB R**	CGCTTCTAAATTGGCACCGC	Reverse primer for double stranded probe for *lmo0945* mRNA
**lmo0946 NB F**	ATGAAAAAAGCAATTTTAGATCGG	Forward primer for double stranded probe for *sif* mRNA
**lmo0946 NB R**	GATGAATCGAGCTTTTCTTTG	Reverse primer for double stranded probe for *sif* mRNA
**lhrC5 NB F**	ATAAGCTAACAACAAGCAAAACATTTTCATTTCTTTCCC TTTTTA**GAATGGAAATCCCAAACTCCC**	Forward primer for double stranded probe for *lhrC5* mRNA. Anneals with lhrC5 NB R (bold).
**lhrC5 NB R**	AAAAAAACTAGTGCGGAAAAAGGGAGTAAACCGCACTA GCTAAAA**GGGAGTTTGGGATTTCCATTC**	Reverse primer for double stranded probe for *lhrC5* mRNA. Anneals with lhrC5 NB F (bold).
**16S agarose NB F**	GTGCATTAGCTAGTTGGTAG	Forward primer for double stranded probe for 16S rRNA
**16S agarose NB R**	CAACAGTACTTTACGATCCG	Reverse primer for double stranded probe for 16S rRNA
**Fri probe**	GCTTCCGCCGATTGCTAGTAAACGTTCTGCTAC	Single stranded probe for *fri* mRNA
**16S rRNA probe**	TTGCTTCGAATTAAACCACATGCTCCACCGCTT	Single stranded probe for 16S rRNA
	**EMSA RNA, structure probing and translation *in vitro* analysis**	

**EMSAfriF 1**	**TAATACGACTCACTATAGGG**AAGATAAATAATGAGAAAATGGTTATAGATG	Forward primer for synthesis of *fri* mRNA_TSS1 DNA with T7 promoter (bold) to be transcribed into RNA.
**EMSAfriF 2**	**TAATACGACTCACTATAGGG**ATGGTTTCCACACTGGACAT	Forward primer for synthesis of *fri* mRNA_TSS2 DNA with T7 promoter to be transcribed into RNA.
**EMSAfriF 3**	**TAATACGACTCACTATAGGG**AAGTGATAATCATTATAAAATAGGAGGAAG	Forward primer for synthesis of *fri* mRNA_TSS3 DNA with T7 promoter to be transcribed into RNA; also for structure probing and *in vitro* translation.
**EMSAfriR**	GTTTAAATTCGCTACTTGGTGATTC	Reverse primer for synthesis of *fri* mRNA_TSS1, *fri* mRNA_TSS2, and *fri* mRNA_TSS3 DNA to be transcribed into RNA; also for structure probing.
**fri R**	AGACAAAAAAACTGTACGAGACGATTAATCCCGCACAGTTTTTATTACTCTCTACTCTAATGGAGC	Reverse primer for synthesis of *fri* DNA to be transcribed into RNA for *in vitro* translation
**PT7LhrC5F**	**TAATACGACTCACTATAGGG**ATAAGCTAACAACAAGCAAAACATTTTCATTTCTTTCCCTTTTTAGAATGGAAATCC	Forward primer for synthesis of *lhrC5* DNA with T7 promoter (bold) to be transcribed into RNA; also for structure probing and *in vitro* translation. Anneals with PT7LhrC5R (underlined).
**PT7LhrC5R**	AAAAAAACTAGTGCGGAAAAAGGGAGTAAACCGCACTAGCTAAAAGGGAGTTTGGGATTTCCATTCTAAAAAGGG	Reverse primer for synthesis of *lhrC5* DNA with T7 promoter (bold) to be transcribed into RNA; also for structure probing and *in vitro* translation. Anneals with PT7LhrC5F (underlined).
**PT7LhrC5_modF**	**TAATACGACTCACTATAGGG**ATAAGCTAACAACAAGCAAAACATTTTCATTTCTTTAAGTTTTTAGAATGGAAATCC	Forward primer for synthesis of *lhrC5*_mod DNA with T7 promoter (bold) to be transcribed into RNA; also for structure probing and *in vitro* translation. Anneals with PT7LhrC5_modR (underlined).
**PT7LhrC5_modR**	AAAAAAACTAGTGCGGAAAAAGGGAGTAAACCGCACTAGCTAAAAGGGAGTTTGGGATTTCCATTCTAAAAACTT	Reverse primer for synthesis of *lhrC5*_mod DNA with T7 promoter (bold) to be transcribed into RNA; also for structure probing and *in vitro* translation. Anneals with PT7LhrC5_modF (underlined).
**PT7LhrC4F**	**TAATACGACTCACTATAGGG**ATAAGCTAACAACAAACAAAACATTTTCATTCTTCTCCCCCCTTTTAGAATGAAAATCC	Forward primer for synthesis of *lhrC4* DNA with T7 promoter (bold) to be transcribed into RNA. Anneals with PT7LhrC4R (underlined).
**PT7LhrC4R**	AAAAAAACCGATGCGGAAAAGGGAGTAAACCGCATCGGTCAAAAAAAGGGAGTTTGGGATTTTCATTCTAAAAGGG	Reverse primer for synthesis of *lhrC4* DNA with T7 promoter (bold) to be transcribed into RNA. Anneals with PT7LhrC4F (underlined).

FULL SCAN OF THE ORIGINAL GELS

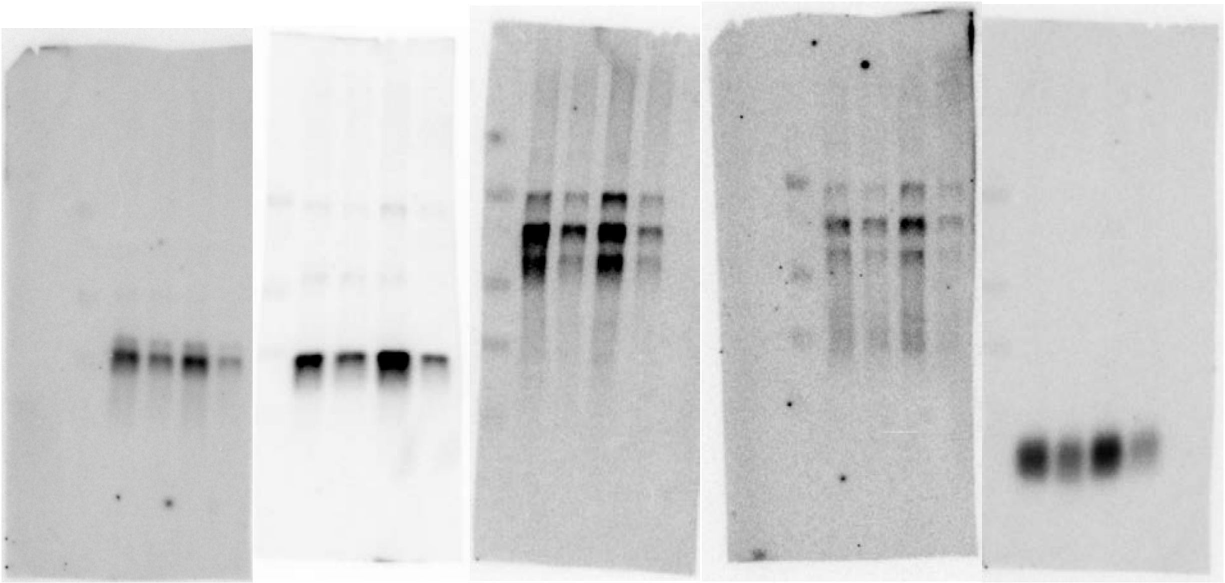

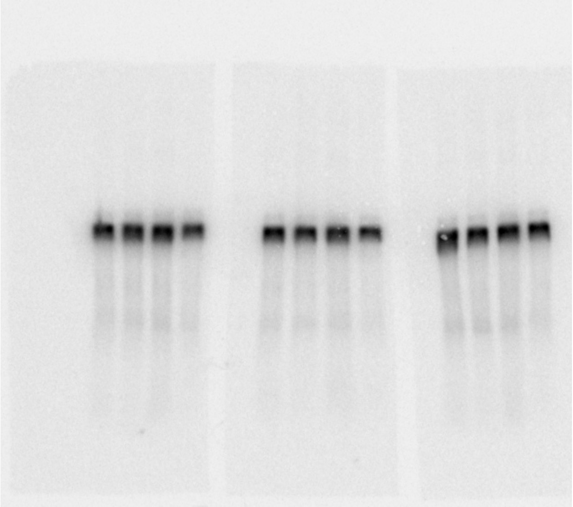

Figure 1. Upper panel (left to right): Northern blots probed for *fri*, *lmo0944*, *lmo0945*, *sif*, and LhrC5. For the *lmo0945* and *sif* blots, the membranes were stripped and subsequently reprobed for LhrC5 and *fri*, respectively.

Lower panel: Northern blots probed for 16S rRNA.

Fig 2B

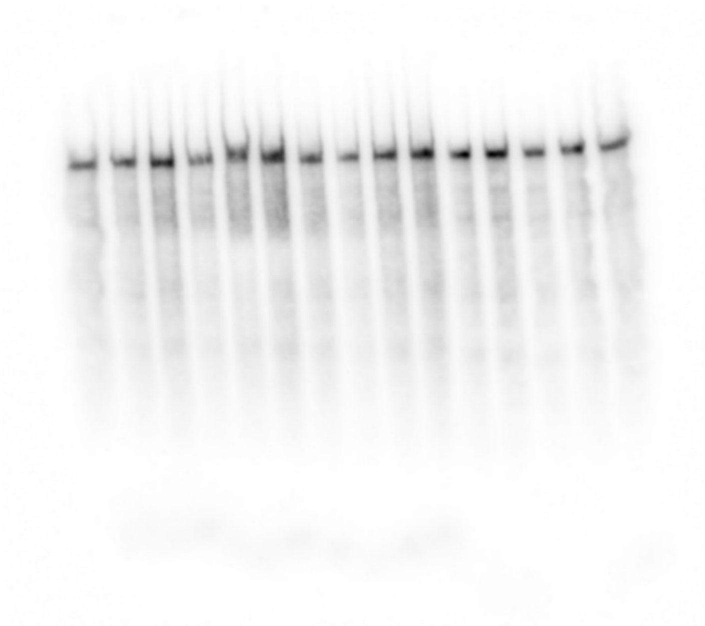

Fig 2C

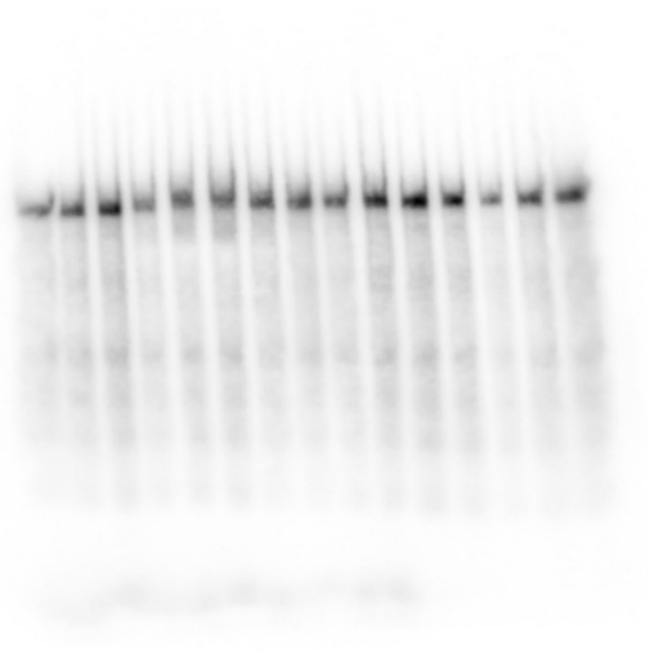

Figure 2

Fig 3A

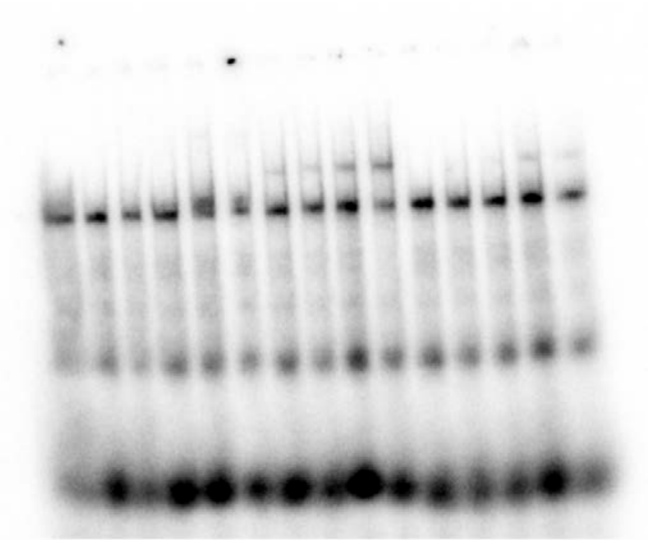

Fig 3C

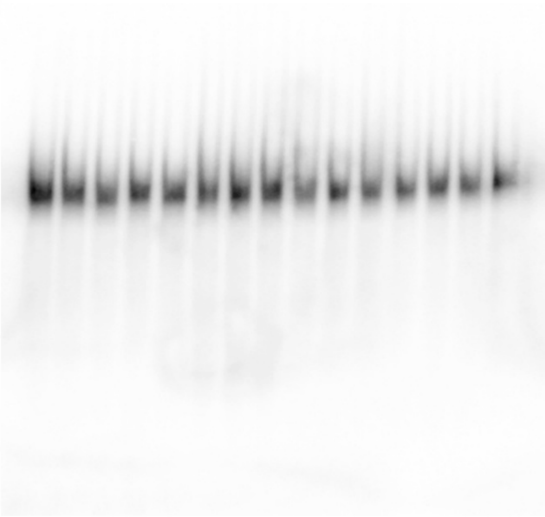

Fig 3C

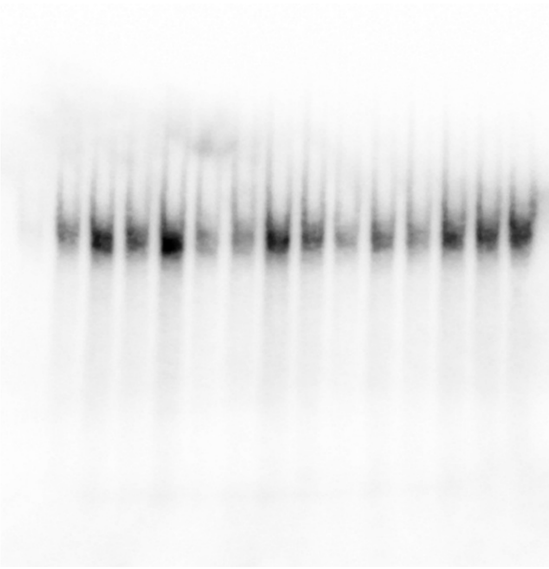

Figure 3

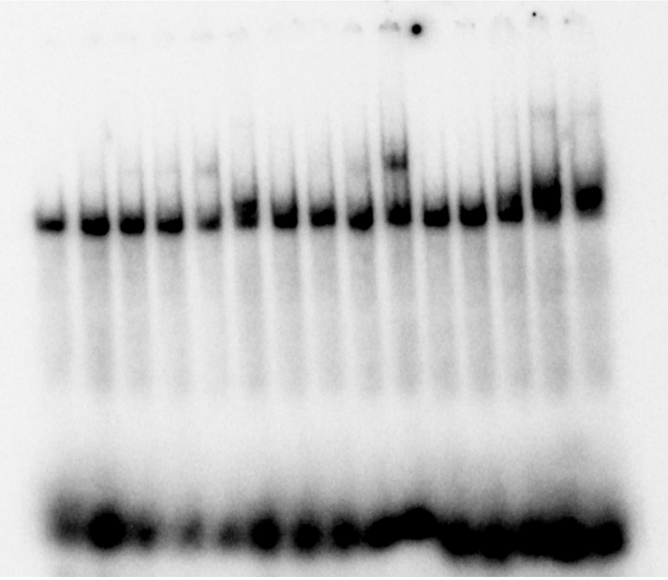

Figure 4

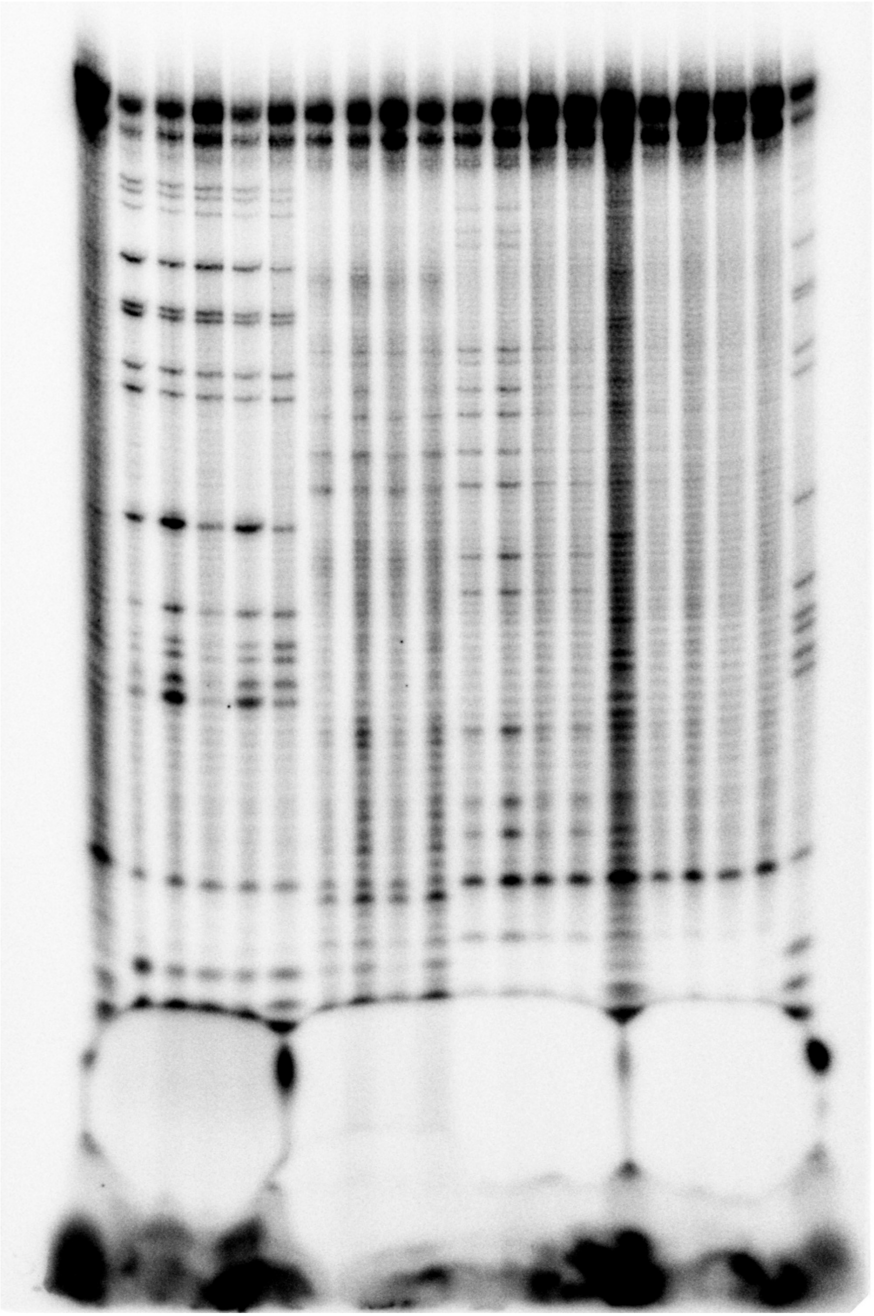

Figure 5

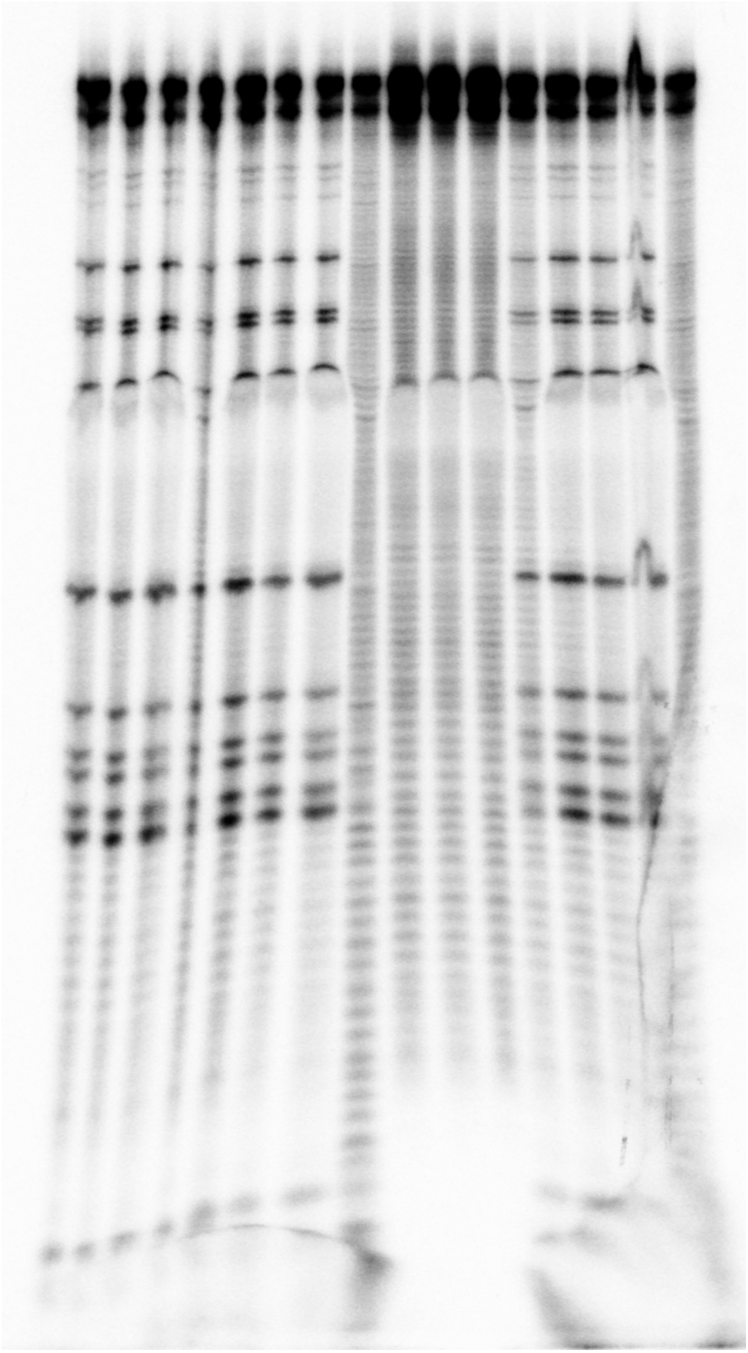

Figure 6

Fig 8A

Fig8B

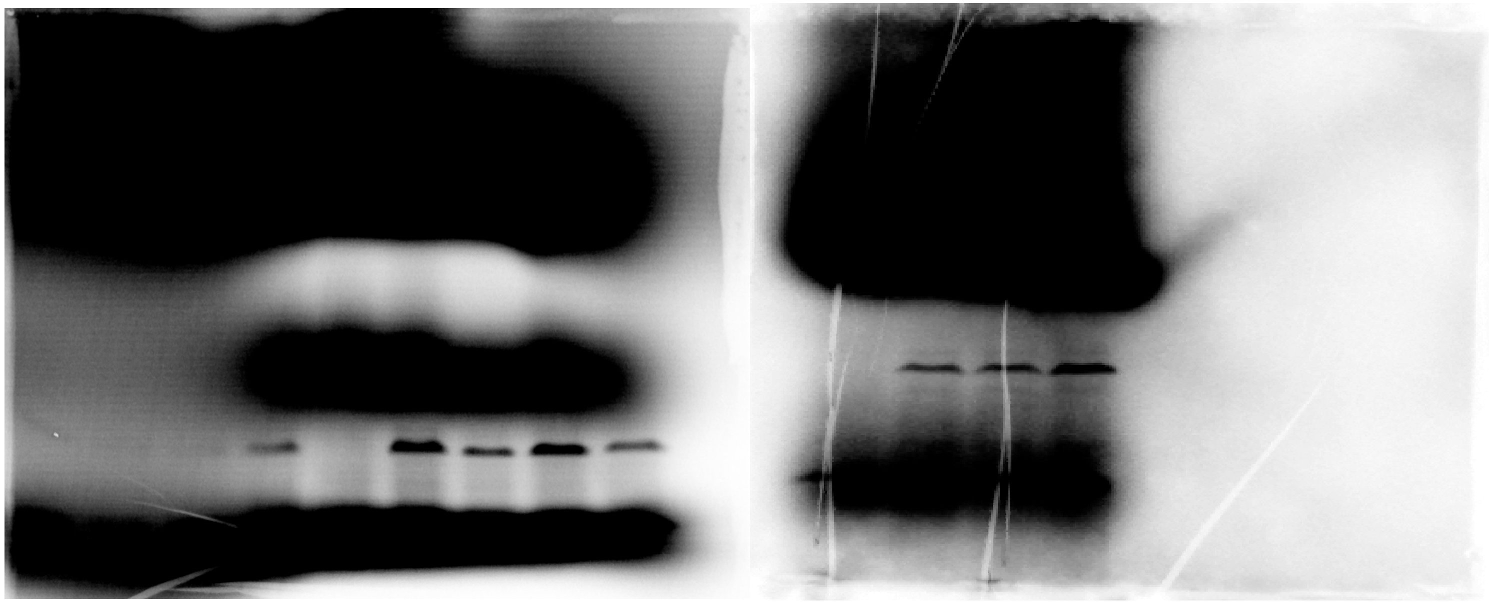

Figure 8

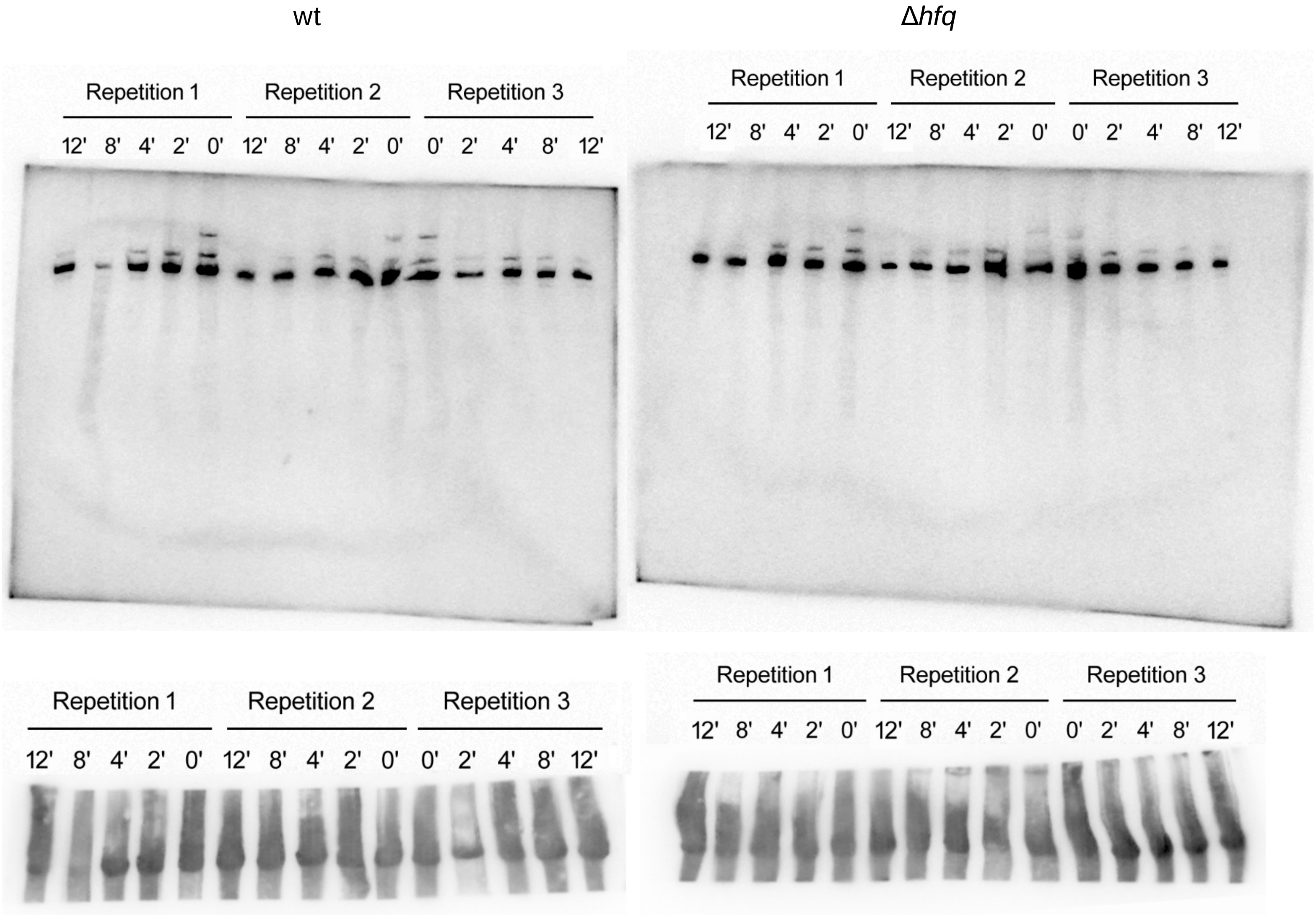
wt
Δhfq
Repetition 1
Repetition 2
Repetition 3
12' 8' 4' 2' 0' 12' 8' 4' 2' 0' 0' 2' 4' 8' 12'
Repetition 1
Repetition 2
Repetition 3
12' 8' 4' 2' 0' 12' 8' 4' 2' 0' 0' 2' 4' 8' 12'
Repetition 1
Repetition 2
Repetition 3
12' 8' 4' 2' 0' 12' 8' 4' 2' 0' 0' 2' 4' 8' 12'
Repetition 1
Repetition 2
Repetition 3
12' 8' 4' 2' 0' 12' 8' 4' 2' 0' 0' 2' 4' 8' 12'

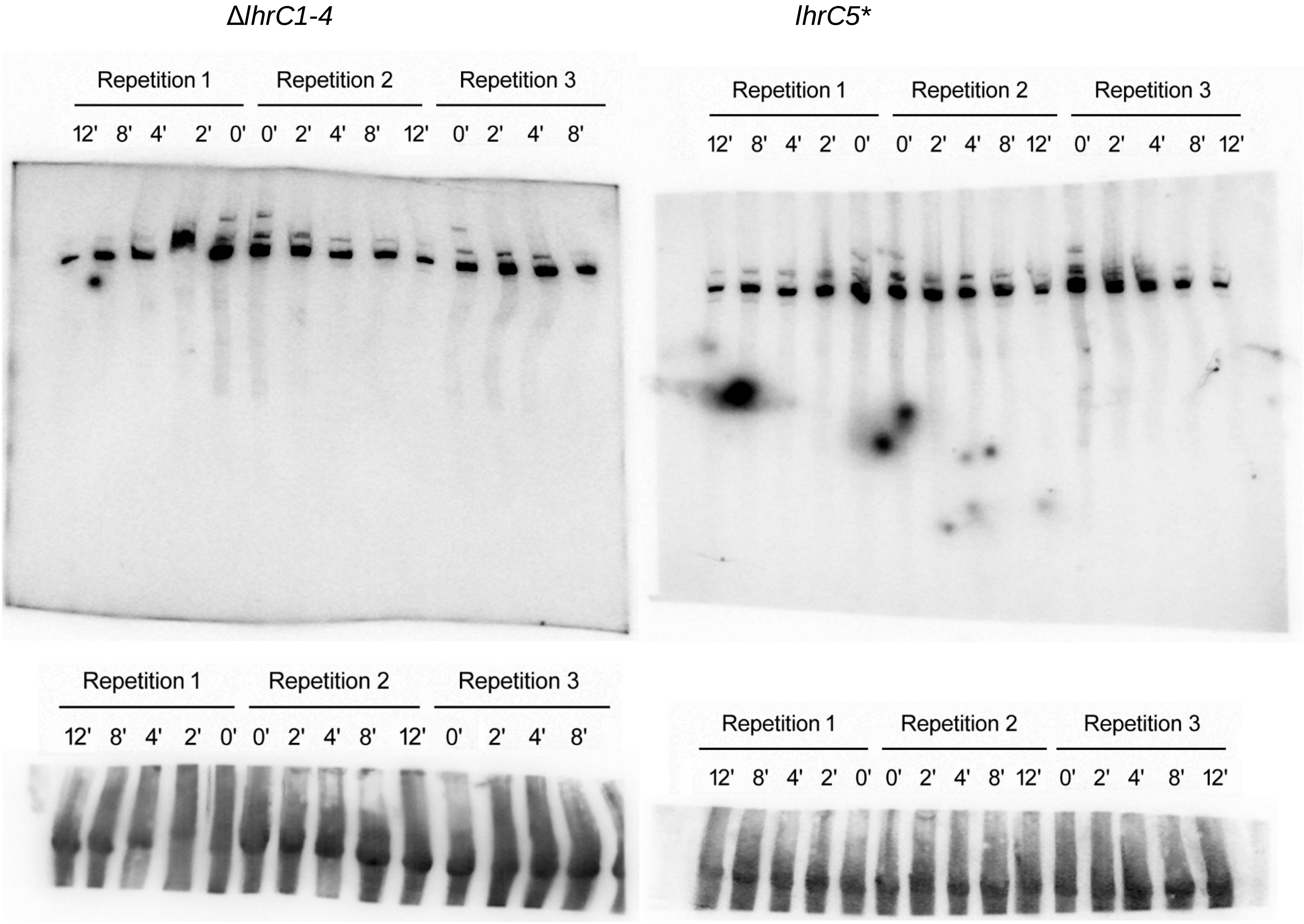
ΔlhrC1-4
lhrC5*
Repetition 1
Repetition 2
Repetition 3
12' 8' 4' 2' 0' 0' 2' 4' 8' 12' 0' 2' 4' 8'
Repetition 1
Repetition 2
Repetition 3
12' 8' 4' 2' 0' 0' 2' 4' 8' 12' 0' 2' 4' 8' 12'
Repetition 1
Repetition 2
Repetition 3
12' 8' 4' 2' 0' 0' 2' 4' 8' 12' 0' 2' 4' 8'
Repetition 1
Repetition 2
Repetition 3
12' 8' 4' 2' 0' 0' 2' 4' 8' 12' 0' 2' 4' 8' 12'

Figure 9

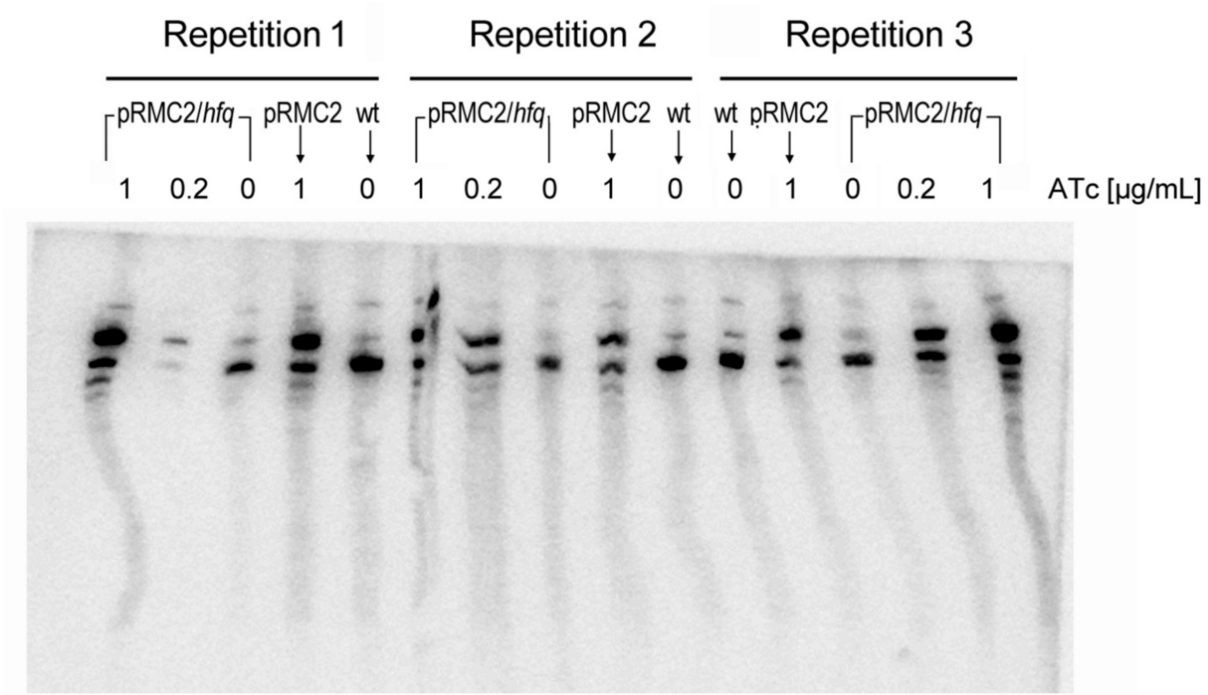

Repetition 1
Repetition 2
Repetition 3
pRMC2/hfq
pRMC2
wt
pRMC2/hfq
pRMC2
wt
wt
pRMC2
pRMC2/hfq
1
0.2
0
1
0
1
0.2
0
1
0
0
1
0
0.2
1
ATc [µg/mL]

Figure 10

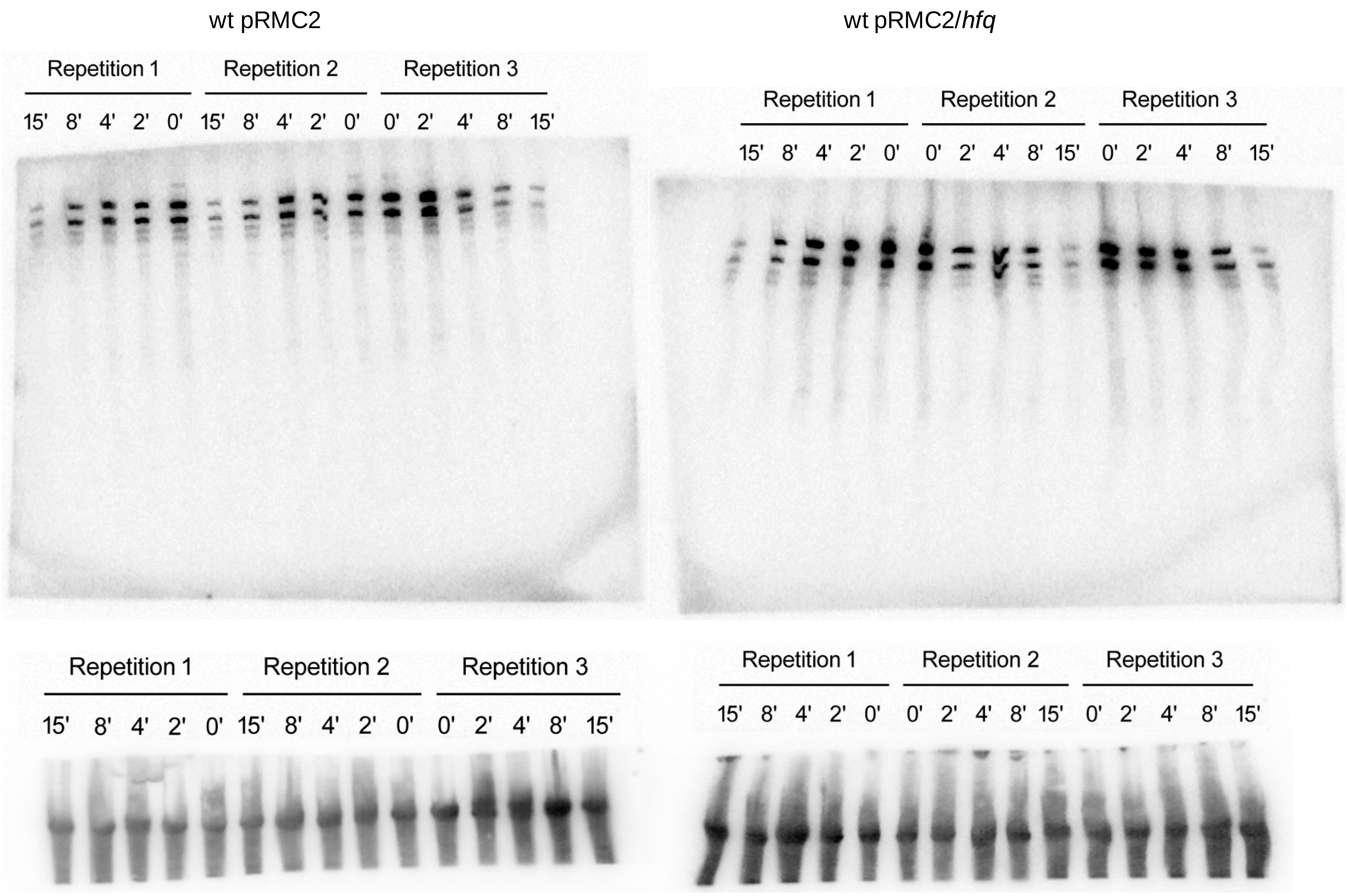
wt pRMC2
wt pRMC2/*hfq*
Repetition 1
Repetition 2
Repetition 3
15' 8' 4' 2' 0' 15' 8' 4' 2' 0' 0' 2' 4' 8' 15'
Repetition 1
Repetition 2
Repetition 3
15' 8' 4' 2' 0' 0' 2' 4' 8' 15' 0' 2' 4' 8' 15'
Repetition 1
Repetition 2
Repetition 3
15' 8' 4' 2' 0' 15' 8' 4' 2' 0' 0' 2' 4' 8' 15'
Repetition 1
Repetition 2
Repetition 3
15' 8' 4' 2' 0' 0' 2' 4' 8' 15' 0' 2' 4' 8' 15'

Figure 11
